# A Review of Gut Microbiota Dynamics: From Healthy Gestation to Gestational Diabetes in Human and Mouse Models

**DOI:** 10.3390/biomedicines14030707

**Published:** 2026-03-18

**Authors:** Dat Da Ly, Bryony A. McNeill, Kathryn Aston-Mourney, Leni R. Rivera

**Affiliations:** School of Medicine, IMPACT, The Institute for Mental and Physical Health and Clinical Translation, Deakin University, Geelong, VIC 3220, Australia; s223518527@deakin.edu.au (D.D.L.); bryony.mcneill@deakin.edu.au (B.A.M.); k.astonmourney@deakin.edu.au (K.A.-M.)

**Keywords:** gut microbiota, early pregnancy, late pregnancy, gestational diabetes

## Abstract

Over the past decades, gut microbiota has emerged as a critical modulator of human health and disease. Pregnancy involves substantial microbiota remodelling that influences offspring development, yet mechanisms linking maternal microbiota changes to gestational diabetes mellitus (GDM) remain unclear. The current literature lacks a comprehensive synthesis of pregnancy microbiota dynamics across healthy gestation to GDM, comparative human–murine analysis, and pregnancy-specific mechanistic frameworks distinct from type 2 diabetes models. This narrative review comprehensively synthesised evidence on gut microbiota composition in healthy pregnancy and GDM (2005–2025, NCBI PubMed) to identify convergent signatures and articulate pregnancy-specific mechanisms. Early pregnancy microbiota resembles non-pregnant individuals, whereas late pregnancy exhibits increased lactic acid-producing bacteria and reduced Firmicutes-to-Bacteroidetes (F/B) ratios. GDM exhibits pathological dysbiosis with elevated F/B ratios and reduced *Bifidobacterium*. Critically, GDM butyrate-producer patterns diverge from type 2 diabetes, suggesting pregnancy-specific mechanisms beyond glucose homeostasis. Despite these insights, methodological heterogeneity and cross-sectional designs constrain definitive conclusions. Longitudinal studies with standardised sequencing are essential to confirm consistent signatures and enable rational design of microbiota-modulating interventions (prebiotics, probiotics, synbiotics, postbiotics, diet) to optimise maternal health, prevent GDM, and support offspring development.

## 1. Introduction

The human gastrointestinal (GI) tract provides a massive interface (250–400 m^2^) between the internal body and external environmental factors [1]. It processes approximately 1–2.5 kg of food daily, breaking it down into absorbable units whilst simultaneously functioning as a barrier against pathogenic invasion [2,3]. These functions are conducted in collaboration with about 100 trillion microorganisms within the gut, collectively known as the gut microbiota [4], which play diverse roles in a range of processes including food and xenobiotic metabolism, antimicrobial protection, and immunomodulation [5].

The gut microbiota is a rich ecosystem that has co-evolved in the human body symbiotically [6,7]. Its primary establishment commences at birth and is shaped by multiple factors including maternal health status, delivery mode, breastfeeding practice, maternal diet, antibiotics and early environmental exposures [8,9]. This early microbial composition established during infancy appears to influence host metabolism and immune development throughout life [8,9,10,11]. Disruptions in normal infant microbial colonisation, termed dysbiosis, have been associated with increased risk of both intestinal disorders and longer-term metabolic and neurological conditions, including type 1 diabetes, obesity, asthma and autism spectrum disorders [12,13,14,15,16,17]. Given that maternal microbiota directly influences infant gut colonisation and has demonstrable long-term health consequences [8,9,10,11], investigations of maternal microbiota dynamics during pregnancy have become increasingly important.

Microbiota research during pregnancy is critical for both metabolically healthy women and those with pregnancy complications such as diabetes. Understanding how diabetes during pregnancy affects the gut microbiota is particularly timely given concerning epidemiological trends showing that, despite public health efforts, the prevalence of pre-existing diabetes is expected to increase due to advancing maternal age at conception, combined with rapidly rising global incidence of type 2 diabetes (T2D) (affecting more than 530 million adults in 2024) [18] and early-onset T2D (diagnosed before age 40) [19]. Meanwhile, the rate of gestational diabetes mellitus (GDM) also continues to rise—currently affecting about 18% of pregnancies in Australia and 14% worldwide [20,21]. Both pre-existing diabetes and GDM are known to increase the likelihood of infants having an increased risk of obesity and type 2 diabetes later in life [22,23]. These conditions are also associated with gut dysbiosis, which has shown significant links to health outcomes not only in infancy but also well into adulthood [24,25,26,27]. Despite the critical health implications of understanding the pregnancy microbiome, variations in geographic regions, dietary habits, gestational stages, sequencing depth and resolution have limited our understanding of the microbial profiles of pregnant individuals, either with or without diabetes.

In non-pregnant adults, studies exploring the relationship between metabolic syndrome and gut microbiome have expanded therapeutic opportunities and clinical significance. A meta-analysis of 15 randomised controlled trials demonstrated that probiotic supplementation significantly reduced HbA1c, fasting blood glucose, and HOMA-IR (homeostatic model assessment for insulin resistance) compared to placebo in individuals with T2D [28]. Another meta-analysis of 14 randomised controlled trials showed that combining metformin with probiotics produced greater reductions in fasting blood glucose and HbA1c, and lower gastrointestinal side effects, compared to metformin monotherapy alone in adults aged 45–65 with T2D [29]. More directly demonstrating causality, allogenic faecal microbiota transplantation from lean donors to male recipients with metabolic syndrome significantly improved insulin sensitivity and increased colonisation by commonly recognised beneficial bacteria such as *Akkermansia* and short chain fatty acid (SCFA)-producing *Eubacterium* within 6 weeks [30,31]. In animal studies, Backhed and colleagues discovered that the colonisation of *Clostridium scindens* on germ-free female mice increased deoxycholic acid, which was positively correlated with HOMA-IR, HbA1c, triglycerides, and negatively associated with high-density lipoprotein, indicating impaired glucose and lipid metabolism [32]. Additionally, the transfer of unfractionated cecal microbiota—predominantly containing opportunistic bacteria *Bacteroides* and *Clostridium*—from conventionally raised to germ-free mice induced body fat accumulation and insulin resistance within 2 weeks, despite reduced food intake [33]. These findings directly demonstrate that dysbiotic microbiota composition can drive metabolic dysfunction independent of dietary intake, highlighting the pathogenic potential of specific bacterial taxa on host metabolism.

Despite the well-established role of the gut microbiome in metabolic health and disease pathogenesis, substantial evidence gaps limit clinical translation and precision therapeutic strategies in pregnancy. The current literature lacks: (1) comprehensive synthesis of pregnancy microbiota dynamics across the full spectrum from healthy gestation to gestational diabetes, integrating both physiological adaptations and pathological remodelling; (2) comparative analysis of human and murine pregnancy microbiota, which would bridge translational mechanistic research with clinical insights; (3) identification of converging microbiota signatures across heterogeneous populations and methodologies, which would establish robust biomarkers despite study variability; and (4) pregnancy-specific mechanistic frameworks distinct from type 2 diabetes models, recognising the unique physiological and metabolic challenges specific to gestation, placental development, and foetal nutrient demands.

Geographic variation, diverse sequencing methodologies, limited longitudinal data, and the tendency to extrapolate directly from T2D models further complicate synthesis of existing evidence and hinder the development of pregnancy-specific therapeutic strategies. Therefore, this narrative review synthesises existing literature on how the gut microbiota shifts through different stages of pregnancy in diverse populations under both healthy and diabetic conditions. By identifying convergent microbiota signatures across heterogeneous studies, integrating human and animal model evidence, and articulating pregnancy-specific mechanistic insights, this review establishes the evidence base necessary for developing clinical strategies, nutritional guidelines, and microbiota-targeted interventions tailored to maternal care and GDM prevention.

## 2. Literature Search Strategy

This narrative review synthesised all available evidence on gut microbiota composition during healthy pregnancy and gestational diabetes in human and mouse models. A structured search of the NCBI PubMed database was performed to identify studies published between January 2005 and February 2025 using keywords and combinations: “gut microbiome”, “gut microbiota”, “gut bacteria”, “pregnancy”, “trimester 1”, “trimester 3”, “gestational diabetes”, “GDM”, “human”, “mouse”, “murine”.

All studies reporting gut microbial composition during pregnancy or GDM were included, regardless of sequencing platform (16S rRNA or whole genome metagenomics) or study location. Given the high variability of reported taxa across studies and the heterogeneity of reported microbial signatures across cohorts and platforms, comprehensive data synthesis was prioritised over formal inclusion and exclusion criteria. Conference abstracts, studies unrelated to pregnancy or GDM and microbiome analyses only focusing on oral or vaginal microbial composition or discussing microbial metabolites but lacking sequencing data of microbial composition in the gut were excluded. To ensure completeness, database searches were supplemented with reference list screening of included studies to identify additional relevant publications. Consequently, a total of 87 studies were included summarising gut microbial compositional across different stages of healthy pregnancy and gestational diabetes in both human and murine models.

## 3. Overview and Establishment of the Neonatal Microbiome

A dynamic and complex ecosystem comprising bacteria, archaea, eukaryotic microbes and viruses, the gut microbiota contains as many cells as the number of human cells in the body [34]. Its microbial genome (so-called “microbiome”) represents 150-fold more genetic varieties than the entire human genome [8,35,36,37,38]. The compilation of the MetaHit (Metagenomics of the Human Intestinal Tract), Human Microbiome Project and Chinese cohort, providing the most comprehensive and intercontinental view on the human microbial repertoire, revealed twelve bacterial phyla and one archaeal taxon (Euryarchaeota) of Prokaryotes as commensals or pathogens in human beings [39,40]. Meanwhile, according to rRNA sequencing and GutFeelingKB, a reference of curated metadata for healthy gut microbiome, healthy human gut microbiota can be taxonomically classified into 8 phyla, 18 families, 38 orders and 59 genera, representing more than 7000 strains—belonging to at least 1000 specific phylogenetic types (or “phylotypes”) [41,42,43]. The eight gut microbial phyla, in order of decreasing phylogenetic abundance, are: Bacillota and Bacteroidota (formerly known as Firmicutes and Bacteroidetes), Actinomyceota and Pseudomonadota (formerly named Actinobacteria and Proteobacteria), Verrucomicrobia, Fusobacteria, Synergistetes and Euryarchaeota [44].

The GI tract of the foetus was initially believed to be sterile until the first microbial colonisation at birth [3,38]. However, this belief has been challenged by several findings over the past decades which have demonstrated the existence of bacterial DNA or certain SCFAs in the first-pass meconium, placenta, umbilical cord blood and amniotic fluid in healthy pregnancy [45,46,47,48,49,50,51,52,53]. The placenta has been identified as a niche for commensal species such as Bacillota, Bacteroidota, Pseudomonadota, and Fusobacteriota, and largely resembles the mother’s oral microbiota [52]. The observed similarities among the microbial profiles of the mother’s mouth, placenta, amniotic fluid, and first-pass stool suggest the bacteria cross the placenta—a selective barrier between foetus and mother—and their potential translocation to the developing gastrointestinal tract [45]. This underscores the critical role of the maternal microbiota in shaping the initial establishment of the infants’ microbiome, regardless of differing views on its origin.

Although it is now known that colonisation commences during intrauterine life, the period immediately after birth plays a significant role in determining the initial gut microbial composition. Mode of delivery is a major factor influencing the initial gut microbial composition [3,38,54]. The gut microbiota of vaginal delivered newborns has been shown to inherit the ecosystem of their mother’s vagina, which typically includes Bacteroidota species, especially *Lactobacillus*, *Prevotella* genera or *Sneathia* genera under the Fusobacteriota phylum [54,55]. Meanwhile, the gut microbiota from newborns delivered via caesarean section is dominated by *Clostridium*, *Staphylococcus*, *Corynebacterium*, and *Propionibacterium* genera—which resembles the microbiota of maternal skin or the hospital environment [55,56,57]. Caesarean-delivered infants also had less diverse species and experienced delayed colonisation of Bacteroidota microorganisms compared to babies delivered vaginally [55,56]. Therefore, the establishment of the infants’ gut microbiome can vary substantially and has the potential to significantly influence ongoing health in childhood and adulthood.

## 4. Baseline Gut Microbiota Composition: Non-Pregnant Individuals

### 4.1. Dominant Bacterial Phyla in Healthy Adults

Establishing the baseline gut microbiota composition in non-pregnant individuals is essential for understanding how it adapts and changes during pregnancy. While microbiota data specifically from non-pregnant women of reproductive age remain limited, data from healthy non-pregnant adults provide a valuable reference point for understanding pregnancy-related microbial shifts. While differences in the core microbiome across continents and ethnicities should be acknowledged, it is well recognised that Firmicutes, Bacteroidetes, Actinobacteria, and Proteobacteria are the dominant phyla in healthy human gut communities [3,58,59,60,61]. In healthy adults, the gut microbiota is dominated by Firmicutes (40–70%), followed by Bacteroidetes (20–45%) and Actinobacteria (1–10%), though these proportions may vary with diet, geography, and other factors [39,44,61,62,63,64,65], which is consistent with reports specifically describing the proportions in healthy women of child-bearing age, particularly Firmicutes (50–70%), Bacteroidetes (20–35%), Actinobacteria (2–9%), and Proteobacteria (3–4%) [66,67].

### 4.2. Lower Taxonomic Ranks: Dominant Genera and Enterotypes

At the genus level and below, specific dominant taxa have been consistently identified across independent studies. Key genera include *Faecalibacterium*, *Clostridium*, *Lachnospiraceae*, *Roseburia*, *Ruminococcus* (under Firmicutes); *Bacteroides, Prevotella*, *Alistipes* (under Bacteroidetes); and *Bifidobacterium* (under Actinobacteria) [39,44,61,62,63,64]. Among these, studies from different cohorts have reliably identified key taxa that underpin three major gut microbiota enterotypes representing distinct microbial community structures: *Ruminococcus*-dominant, *Bacteroides*-dominant, and *Prevotella*-dominant networks [44,68,69,70,71]. Whilst these enterotypes likely reflect stable, long-term microbial configurations, they can be shaped by individual factors including diet and host genetics. When interpreting microbiota findings across studies, several methodological factors warrant careful consideration. Cross-study differences in participant age, geographic location, dietary background, and sequencing methodologies can substantially influence reported microbiota composition. Additionally, faecal samples serve as a convenient proxy for gut microbiota but do not fully represent the complexity of the entire gastrointestinal tract, which exhibits compositional variation both axially (mucosal vs. luminal communities) and longitudinally (proximal vs. distal regions) [72,73]. These limitations underscore the importance of cautious interpretation when generalising findings across populations and study designs.

## 5. Gut Microbiota Composition in Early Pregnancy

The gut microbiota is shaped by multiple factors, including host genetics, age, diet, antibiotic use, and geography [74,75,76,77]. Alterations in its composition are also commonly reported in inflammation-related disorders such as Crohn’s disease, inflammatory bowel disease (IBD), allergies, as well as in conditions linked to host adiposity and insulin resistance [78,79,80,81,82]. Therefore, in response to substantial metabolic and inflammatory changes during a healthy pregnancy to support implantation, placentation, and foetal growth, corresponding shifts in the gut microbiota are expected [83]. There is limited research directly comparing the gut microbiota of healthy women in the first trimester of pregnancy with the microbial profile before conception. Most existing studies examine changes from early to late pregnancy rather than comparing to the non-pregnant state, and many focus on women with high body mass index (BMI) and/or varying GDM status. Although data are limited, early pregnancy (from the first to early second trimester, before week 20) is typically characterised by a proinflammatory state, with or without insulin resistance [84,85]. This stage likely exerts a smaller influence on gut microbiota composition compared to later pregnancy, when pronounced metabolic changes such as increased insulin resistance and anti-inflammatory adaptations become more evident (Table 1). According to the microbial analysis conducted by Koren et al. in pregnant women across trimesters, although late pregnancy showed significant shifts, early pregnancy microbiota resembled that of non-pregnant individuals, with comparable β-diversity and differences in Firmicutes and Bacteroidetes abundance [86].

### 5.1. Phylum Level Composition in Early Pregnancy

Altered relative abundance of Firmicutes and Bacteroidetes has been observed in early pregnancy [86]; though direct evidence linking their abundance to specific physiological changes during early pregnancy remains limited. In healthy non-pregnant adults, Firmicutes has been positively associated with increased adiposity and triglyceride levels, whereas Bacteroidetes shows a negative association [87,88]. This relationship may partly explain findings from healthy early pregnancies, where weighted UniFrac analysis revealed that first-trimester samples clustered toward a higher Firmicutes gradient and a lower Bacteroidetes gradient compared to non-pregnant controls [86]. However, a case–control study of 98 healthy women at gestational weeks 10–15 reported comparable relative abundances of these phyla [25]. Such discrepancies highlight ongoing controversies regarding F/B ratios in early pregnancy. These inconsistencies are further complicated when overweight (non-GDM) pregnancies are included, in which greater variation in F/B ratio was observed. For instance, at gestational week 16, overweight (non-obese, non-GDM) women exhibited a F/B ratio of approximately 3:1 [87]. In contrast, Mokkala et al. found a higher proportion of Bacteroidetes compared to Firmicutes at week 14 among 270 overweight/obese women (including 203 without GDM onset). This finding is consistent with their earlier research on 60 overweight/obese non-GDM women and the sub-unit value of the F/B ratio in a matched case–control study of 120 healthy controls at week 10 [89,90,91]. Overall, phylum-level composition during early pregnancy closely resembles that of non-pregnant individuals; particularly, Firmicutes, Bacteroidetes, Actinobacteria, and Proteobacteria remain predominant during the first trimester, with Firmicutes and Bacteroidetes together accounting for over 80% of total microbial abundance, Actinobacteria 2–10% and Proteobacteria roughly 2% [25,61,86,87,89,90,91,92,93,94,95,96,97,98]. However, the relative dominance hierarchy among these phyla, particularly the ranking of Firmicutes over Bacteroidetes, or Actinobacteria over Proteobacteria, is not consistently reported across first trimester studies, reflecting underlying variability across cohorts.

### 5.2. Lower Taxonomic Ranks and Enterotype Patterns in Early Pregnancy

At the genus level, microbial hierarchies also mirror those of non-pregnant cohorts: *Bacteroides*, *Prevotella*, and *Alistipes* dominate within Bacteroidetes, while *Faecalibacterium*, *Blautia*, *Agathobacter*, and *Roseburia* dominate within Firmicutes, establishing Clostridia as the leading class both before and during early pregnancy [25,61,86,87,89,90,91,92,93,94,95,96,97,98]. Among all genera, *Bacteroides* consistently represents the most abundant taxon, followed by *Faecalibacterium*, *Prevotella*, and *Blautia* [25,61,92]. These genera show broad consistency across geographic regions, with the notable exception of *Prevotella*, which exhibits marked variation between U.S. and Chinese cohorts and even among different Chinese populations, driven primarily by dietary differences and host genetics [25,61,92,93]. In normoglycemic pregnancies, the gut microbiota is characterised by increased abundance of Prevotellaceae, Fusobacteriales, and Sutterella [92]. Intriguingly, later GDM onset correlates negatively with *Prevotella* abundance and serum cholesterol levels [92], suggesting a potential protective role for *Prevotella* in early pregnancy.

Analysis by Xiao et al. identified three distinct clusters from gut microbial analysis among 1715 pregnant women worldwide: one *Firmicutes*-dominated cluster and two dominated by *Bacteroides* and *Prevotella*, respectively [61]. These patterns align with enterotypes observed in non-pregnant populations [44]. Specifically, in the first trimester, cluster 1 was enriched in Bacteroidetes (>50%), cluster 2 featured *Prevotella* (~50%) and *Bacteroides* (~12%), and cluster 3 was *Firmicutes*-dominated, with *Faecalibacterium* (~18%) and unclassified *Lachnospiraceae* (~30%). These findings reinforce the concept of a relatively stable gut microbial structure during the transition from non-pregnant to early gestation, despite some variation in Firmicutes and Bacteroidetes abundance, a feature likely linked to mild metabolic and pro-inflammatory changes with pregnancy onset.

**Table 1:** Gut microbial composition in early pregnancy in women without GDM.

Year	Country	Sequencing	*n*	Gestational Week	Status	Microbial Composition
2012 [86]	Finland	16S rRNA (V1-V2)	91	~13.84	2 *	Relative abundance: F ~22–88%, B ~5–70%, A ~5%, P ~1%
2016 [87]	Australia	16S rRNA (V6-V8)	44	16	3 *	Relative abundance: F ~58–81% F/B ratio ~3:1
2017 [90]	Finland	16S rRNA	60	~12.9	3 *	Relative abundance: F ~43.2%, B ~50.5%, A ~1.2%, P ~3.2% Abundant genera: *Faecalibacterium*, *Oscillospira*, *Blautia*, *Lachnospira*, *Ruminococcus*, *Coprococcus*, *Bacteroides*, *Prevotella*, *Parabacteroides*, *Sutterella*
2019 [94]	Israel	16S rRNA (V4)	35	12–14	1 *	Abundant genera: *Faecalibacterium*, *Blautia*, *Ruminococcus*, *unclassified Lachnospiraceae*, *Bacteroides*, *Prevotella*, *Bifidobacterium*
2020 [91]	Finland	Metage- nomics	203	~13.9	3 *	Relative abundance: F ~37.9%, B ~56.9%, A ~1.7%, P ~2.5%
2020 [95]	China	16S rRNA (V3-V4)	103	~9	1 *	Relative abundance: F ~40%, B ~50%, A ~2%, P ~3%
2020 [25]	China	16S rRNA (V4)	98	10–15	1 *	Relative abundance: F ~45%, B ~47%, A ~2%, P ~5% Abundant genera: *Faecalibacterium*, *Ruminococcus*, *Roseburia*, *Agathobacter*, *Bacteroides*, *Prevotella*, *Alistipes*
2020 [97]	China	16S rRNA (V3-V4)	45	Trimester 1	1 *	Relative abundance: F ~47–56%, B ~36–45%, A ~1–3%, P ~2–4%
2022 [92]	Czechia	16S rRNA (V3-V4)	22	Trimester 1	1 *	Abundant class: Clostridia, Bacilli, Negativicutes, Bacteroidia, γ-Proteo-bacteria
2023 [98]	Malaysia	16S rRNA (V3-V4)	12	Trimester 1	1 *	Relative abundance: F ~47%, B ~43%, A ~2%, P ~7%
2023 [89]	China	Whole genome	120	~10.2	1 *	Relative abundance: F ~40%, B ~55%, A ~2.3%, P ~2%
2024 [96]	New Zealand	16S rRNA (V3-V4)	27	12	1 *	Relative abundance: F ~52%, B ~39%, A ~2.3%, P ~3.1% Abundant genera: *Faecalibacterium*, *Oscillospira*, *Roseburia*, *Ruminococcus*, *Coprococcus*, *Bacteroides*, *Prevotella*

* 1: healthy control, 2: varied BMI and GDM onset, 3: overweight/obese without GDM, F: Firmicutes, B: Bacteroidetes, A: Actinobacteria, P: Proteobacteria.

## 6. Gut Microbiota Composition in Late Pregnancy

Beyond the first 20 weeks of gestation, progressive metabolic and immunological adaptations support rapid foetal growth. Elevated circulating concentrations of progesterone, oestradiol, and placental lactogen contribute to the regulation of insulin and promote leptin resistance, facilitating maternal–foetal glucose transfer to meet the increasing demands of the foetus [99]. By the late second and third trimesters, insulin sensitivity can decline by up to 50%, accompanied by low-grade mucosal inflammation. These physiological changes are likely to influence the composition of the commensal gut microbiota during late pregnancy [26,100,101,102].

Gut microbial remodelling in late pregnancy is well documented, with significant β-diversity differences compared to non-pregnant women or early-pregnancy samples across diverse populations in Asia, Europe, the Americas, and Africa (Table 2) [61,86,95,103]. After adjusting for BMI, GDM status and technical factors, the magnitude of β-diversity alteration from first to third trimester were attributed mainly to physiological adaptations of pregnancy [86]. In contrast, α-diversity shows inconsistent patterns with nearly half of studies reporting marked reductions in indices such as Shannon, Faith’s phylogenetic diversity, observed OTUs, or species counts—representing within-sample microbial richness and/or evenness, whilst others find no significant differences [61,86,89,91,93,95,96,97,103,104,105].

### 6.1. Phylum Level Remodelling: Progressive Shifts Toward Dysbiosis Markers

At the phylum level, Actinobacteria is the only group consistently reported to increase as gestation advances [86,94,102], evidenced by frequent enrichment of its dominant genera, *Bifidobacterium* and *Collinsella*, both lactic acid producers [61,93,94,95,106]. Maternal fasting glucose levels have been positively associated with Actinobacteria abundance, while Bifidobacterium, known to promote placental morphogenesis and regulate foetal growth, has been linked to healthy gestational weight gain and increases in abundance in response to elevated circulating progesterone levels during late pregnancy [94,106,107]. Certain *Bifidobacterium* species are recognised as early colonisers of the infant gut, contributing to neonatal immune development [108,109,110,111].

Changes in Firmicutes and Bacteroidetes during healthy pregnancy show less consistency between studies. However, a decline in the F/B ratio during the third trimester is more reproducible and likely represents a physiological adaptation to support foetal glucose demands and gestational weight gain in healthy pregnancies, though whether it constitutes true dysbiosis or predisposes to metabolic complications in at-risk populations remains unclear [89,91,102,104]. The functional significance of this F/B decline becomes evident when compared across metabolic states: higher *Firmicutes* abundance correlates with excessive gestational weight gain, whereas higher Bacteroidetes is associated with normal weight gain [106,112]. This contrasts sharply with obesity-related dysbiosis, which shows elevated *Firmicutes* and reduced *Bacteroidetes* (increased F/B ratio) [62,113,114,115]. Conversely, type 2 diabetes (T2D) is linked to reduced Firmicutes and Clostridia—one of the largest and predominant classes within Firmicutes [116]. Interestingly, a longitudinal case–control study of 120 pairs of matched pregnant controls and women with GDM reported an inverse correlation between F/B ratio and maternal fasting plasma glucose throughout gestation [89]. Thus, despite variability in absolute abundances, the consistent decline in F/B ratio during healthy late pregnancy may reflect adaptive sub-phylum shifts (e.g., increased Bacteroidetes, decreased Firmicutes) that support normal physiological weight gain and glucose metabolism. Proteobacteria, including Enterobacteriaceae, frequently implicated in inflammation-related disorders such as Crohn’s disease, IBD, colorectal cancer, and obesity [117,118,119,120], shows no consistent pattern from early to late pregnancy, but an increasing trend is more well-documented [86,89,97,103,106].

### 6.2. Changes in Lower Taxonomic Ranks: Functional Trade-Offs Between Lactic Acid and Butyrate Producers

At the genus level, randomised controlled trials in metabolically healthy women report increased lactic acid-producing bacteria and decreased butyrate producers as pregnancy progresses [121,122]. For example, *Ruminococcus*, a common butyrate producer within Firmicutes, consistently declines during the third trimester [86,94,103]. Notably, *Ruminococcus gnavus* produces an inflammatory polysaccharide that stimulates TNF-α, and high abundances of certain *Ruminococcus* species have been associated with allergies in children (4–6 years old), inflammatory disorders and mucosal damage in non-pregnant adults [123,124,125,126]. Conversely, *Streptococcus* (Firmicutes), a lactic acid producer, shows consistent increase during pregnancy, similar to *Bifidobacterium* and *Collinsella* (Actinobacteria) [61,86,95,103]. Both genera are considered beneficial for gestational outcomes, as reduced abundance has been associated with spontaneous preterm delivery, as reported in a case–control study from Eastern Norway [127].

Another notable trend is the rise in *Bilophila* throughout gestation [95,102,103], a hydrogen sulfide-producing genus within Desulfovibrionales, typically present at low abundance in healthy guts [128]. *Bilophila* derived from human stool can induce systemic inflammation in pathogen-free mice and impaired glucose tolerance [95,102,103,129,130]. Moreover, *Bilophila* has also been associated with high-fat-diet-induced metabolic dysfunctions in mice [130]. However, a meta-analysis further highlighted the contrasting roles of sulfate-reducing versus lactic acid-producing bacteria in gut inflammation: excessive hydrogen sulfide can provoke intestinal toxicity, whereas lactic acid bacteria enhance nutrient bioavailability, promote beneficial microbiota composition, and help prevent IBD [131]. This nuanced interplay between these bacterial groups is critical for maintaining gut function and barrier integrity during late pregnancy—a period marked by heightened nutritional demands and inflammatory responses.

### 6.3. Mechanistic Insights: Experimental Evidence and Functional Significance

Experimental evidence supports a causal role for microbiota in metabolic adaptations during late pregnancy. Germ-free mice transplanted with third-trimester microbiota exhibited greater weight gain and insulin resistance compared to those receiving first-trimester microbiota [86]. Additionally, multiple studies report correlations between specific taxa and metabolic parameters, including glucose-related markers (FPG, OGTT, HbA1c, HOMA-IR, hyperglycaemia) and adiposity-related measures (HDL, cholesterol, weight gain, dyslipidaemia) [61,89,95,98,102,106,132,133,134]. However, most research has focused on compositional changes, with limited exploration of underlying mechanisms. It remains unclear whether these shifts represent physiological adaptations to meet foetal nutritional demands or protective responses to pregnancy-induced adiposity, insulin resistance, and inflammation. Further mechanistic studies are needed to clarify the functional significance of these taxonomic changes and their contribution to either healthy gestational outcomes or the development of gestational pathologies.

**Table 2:** Gut microbial composition from early to late pregnancy in women without GDM.

Year	Country	Sequencing	*n*	Gestational Week	Status	Microbial Alterations
2008 [112]	Finland	Targeted qPCR *	36	30–35	1 *	↑: *Staphylococcus aureus*, *Bacteroides-Prevotella* group
2010 [106]	Spain	Targeted qPCR *	34	24	1 *	↑: *Bacteroides*, *Bifidobacterium*, *Akkermanisia* ↓: *Staphylococcus*, *C. leptum*, *E. coli*, *Enterobacteriaceae*
2012 [86]	Finland	16S rRNA (V1-V2)	91	33.72	2 *	≠: β diversity ↑: Actinobacteria, Proteobacteria, *Streptococcus*, *S. salivarius*, *L. zeae*, *C. perfringens*, *C. ramosum*, *E. faecalis*, *Propionibacterium*, Enterobacteriaceae ↓: α diversity, *Clostridium*, *Lachnospiraceae*, *Blautia*, *Faecalibacterium*, *Eubacterium rectale*, *Ruminococcaceae*, *R. bromii*, *Subdoligranulum*, Bifidobacteriales
2013 [93]	Norway	Sanger	87	30–40	1 *	*Bifidobacterium* ~2% ↑: *B. adolescentis*, ↓: *B. breve*
2014 [91]	Finland	Metage- nomics	203	~35.2	3 *	↓: α diversity, F/B ratio
2017 [135]	China	Whole genome	81	21–29	1 *	Abundant genera: *Ruminiclostridium*, *Roseburia*, *Lachnospiraceae*, *Bacteroides*, *Prevotella*, *Parabacteroides*
2018 [136]	Brazil	16S rRNA (V4)	42	~33.89	1 *	Abundant genera: *Ruminococcus*, *Eubacterium*, *Bacteroides*, *Prevotella*
2018 [137]	Denmark	16S rRNA (V1-V2)	157	~28.4	1 *	Abundant genera: *Bacteroides*, *Faecalibacterium*, *Prevotella*, *Lachnospiraceae*, *Alistipes*, *Blautia*
2019 [138]	China	16S rRNA (V3-V4)	11	~32,7	1 *	Abundant genera: *Faecalibacterium*, *Roseburia*, *Ruminococcus*, *Bacteroides*, *Prevotella*
2019 [139]	China	Whole genome	26	~40.15	1 *	Abundant genera: *Faecalibacterium*, *Clostridium*, *Eubacterium*, *Roseburia*, *Ruminococcus*, *Blautia*, *Lachnospiraceae*, *Bacteroides*, *Prevotella*, *Alistipes*, *Bifidobacterium*, *Escherichia*
2019 [140]	China	16S rRNA (V3-V4)	16	~25.9	1 *	Abundant genera: *Faecalibacterium*, *Blautia*, *Eubacterium hallii*, *Roseburia*, *Subdoligranulum*, *Phascolarctobacterium*
2019 [94]	Israel	16S rRNA (V4)	35	34–36	1 *	≠: β diversity ↑: Actinobacteria, *Neisseria*, *Blautia*, *Bifidobacterium*, *Collinsella*, *Akkermansia* ↓: Bacteroidetes, *Clostridium*, *Dehalo-* *bacterium*, *Ruminococcus*, Bacteroidales
2020 [95]	China	16S rRNA (V3-V4)	103	~24	1 *	≠: β diversity ↑: F/B ratio, *Blautia*, *Clostridium XI*, *C. sensu stricto*, *Anaerococcus*, *L. incertae sedis*, *Coprococcus*, *Streptococcus*, *Prevotella*, *Bifidobacterium*, *Rothia*, *Collinsella*, *Bilophila* ↓: *Holdemania*, *Clostridium XVIII*, *XIVa*, *XIVb*, *E. incertae sedis*, *Bacteroides*, *Parabacteroides*, *Sphingomonas*, *Acinetobacter*, *Haemophilus*
2020 [141]	China	16S rRNA (V3-V4)	31	~38.5	1 *	Abundant genera: *Faecalibacterium*, *Blautia*, *Coprococcus*, *Bacteroides*, *Bifidobacterium*
2023 [104]	China	16S rRNA (V4)	41	24–28	1 *	Abundant genera: *Faecalibacterium*, *Ruminococcaceae*, *Subdoligranulum*, *Roseburia*, *Streptococcus*, *Lachnospiraceae*, *Bacteroides*, *Bifidobacterium*, *Collinsella*
2023 [133]	China	16S rRNA (V3-V4)	9	Full term	1 *	↑: Coriobacteriaceae, Rhodobacteriaceae
2023 [89]	China	Whole genome	120	~24	1 *	≠: β diversity ↓: α diversity, F/B ratio
2023 [103]	China	16S rRNA (V4)	38–42	T3	1 *	≠: β diversity ↑: Firmicutes, *Clostridium*, *Dialister*, *Lachnospiraceae*, *Streptococcus*, *Enterobacteriaceae*, *Akkermansia*, *Desulfovibrio*, *Bilophila* ↓: Bacteroidetes, *Muribaculaceae S24_7*, *Parabacteroides*, Clostridiales, *Coprococcus*, *Erysipelotrichaceae*, *Megamonas*, *Ruminococcus*, *Megasphaera*
2023 [98]	Malaysia	16S rRNA (V3-V4)	12	Trimester 3	1 *	≠: β diversity
2024 [105]	China	16S rRNA (V4)	31	32–34	1 *	Abundant genera: *Faecalibacterium*, *Bacteroides*, *Prevotella*, *Bifidobacterium*, *Escherichia-Shigella*
2024 [61]	Austria, Brazil, USA, South Africa, China, Italy	Varied	2564 (before batch effect removal)	Trimester 3	2 *	≠: β diversity ↑: *Agathobacter*, *Blautia*, *Dorea Megamonas*, *Fenollaria*, *Prevotella*, *Bifidobacetrium* ↓: α diversity, *Bacteroides*-dominant cluster, *Bacteroides*, *Alistipes*
2024 [96]	New Zealand	16S rRNA (V3-V4)	27	24	1 *	↓: α diversity Abundant genera: *Faecalibacterium*, *Oscillospira*, *Roseburia*, *Ruminococcus*, *Coprococcus*, *Bacteroides*, *Prevotella*,
2024 [102]	China	16S rRNA (V3-V4)	30	~18	1 *	↑: *Bacteroidetes*, *Bilophila* ↓: α diversity, F/B ratio, *Neisseriales*

* Targeted qPCR: non-sequencing method using group or genus-specific primers, 1: healthy control, 2: varied BMI, 3: overweight/obese without GDM, ≠: significantly different, ↑: increase, ↓: decrease.

## 7. Gut Microbiome Composition in Gestational Diabetes

Gut dysbiosis is well documented in T2D, characterised by increased opportunistic pathogens and reduced beneficial taxa [142]. Healthy individuals typically harbour higher levels of butyrate-producing bacteria, whereas T2D shows marked reductions in *Clostridium* species and *Akkermansia muciniphila*—taxa with negative correlations with fasting glucose and HbA1c, suggesting a potential role in disease pathogenesis [143,144,145]. Given that GDM is a pregnancy-specific form of diabetes, similar microbial alterations might be expected; however, GDM-associated microbiota changes remain poorly understood (Table 3). Early evidence from a comprehensive multi-omics study of 394 pregnant women identified gut dysbiosis prior to GDM diagnosis [146], suggesting that pathological microbiota remodelling may precede clinical disease manifestation.

### 7.1. Diversity Patterns: α and β-Diversity as Dysbiosis Markers

Microbial diversity patterns in GDM show contrasting results depending on the reported diversity metric. α-diversity (within-sample richness and evenness) generally shows no significant differences between GDM and healthy controls in most studies, aside from a declining trend during the first four months of pregnancy [61,86,89,91,92,95,132,133,136,137,140,141,146,147]. Conversely, β-diversity differences (between-sample compositional variation) between GDM and non-GDM groups are significant in more than half of studied populations [61,89,92,97,104,134,135,139,140,141,146]. During late pregnancy, women with GDM exhibit similar α and β-diversity trends compared to non-GDM groups, with persistent β-diversity differences (in four of five studies) and significant declines in microbial richness (in three of five studies) [26,61,86,91,104], suggesting potential dysbiosis throughout GDM progression. Importantly, reduced Shannon index and richness have been correlated with higher insulin resistance (HOMA-IR) in T2D cohorts [148], and similar inverse correlations between Shannon index and fasting plasma glucose, triglycerides, and cholesterol have been observed in GDM [89,138]. These findings align with the trends observed in non-pregnant cohorts, for example Chatelier et al., who reported that lower microbial richness in 292 Danish volunteers (including 169 with obesity) was associated with inflammation, insulin resistance, adiposity, and dyslipidaemia [149].

### 7.2. Phylum Level Alterations: Elevated F/B Ratio as a Dysbiosis Marker

At the phylum level, GDM pregnancies frequently show Firmicutes enrichment and Bacteroidetes reduction, resulting in an elevated F/B ratio in either early or late pregnancy compared to the non-GDM group [26,95,97,104,132,134,136,150,151]. This elevated F/B ratio has been proposed as a marker for pathological conditions, including obesity, T2D, and adverse pregnancy outcomes, contrasting sharply with the declining F/B ratio observed in healthy pregnancies [100,101,152,153]. Notably, among women who develop GDM by week 28, a higher F/B ratio was repeatedly detected at week 16 in both obese and overweight women, though Firmicutes abundance was significantly higher in obese than overweight women [87]. Reduced Bacteroidetes abundance was also observed in overweight compared to normal weight pregnancies [106], suggesting an association between early F/B ratio elevation and abnormal weight gain—a potential compounding risk factor. Recently, increased F/B ratio has also been shown to significantly correlate with FPG and 1h-OGTT [154].

Other phyla such as Actinobacteria, Proteobacteria, and Verrucomicrobia show no consistent trends in GDM. Critically, distinctive features of healthy pregnancy such as reduced F/B ratio and increased Actinobacteria (particularly *Bifidobacterium*) are often absent or reversed in GDM [26,61,95,104], suggesting that GDM is characterised by distinct inflammatory and metabolic adaptations driven by heightened insulin resistance that diverge from normal pregnancy physiology.

### 7.3. Lower Taxonomic Rank Alterations: Compensatory Responses and Predictive Biomarkers

At the genus level, alterations in *Faecalibacterium* and *Roseburia*, two major butyrate producers, are inconsistent in GDM and do not mirror the reductions commonly observed in T2D [142,155,156]. Conversely, other butyrate-producing genera, including *Blautia* [97,104,132,137,138,140,147], *Ruminococcus* [90,95,132,136,137], and *Lachnospiraceae* [104,132,136,138], are consistently elevated in GDM compared to healthy controls. This enrichment may represent a compensatory response attempting to maintain metabolic homeostasis. Supporting this hypothesis, a GDM mouse model where live *Lachnospiraceae* and butyrate supplementation improved gut barrier integrity, reduced placental inflammation, and mitigated GDM phenotypes—effects lost when bacteria were heat-inactivated or treated with a butyrate inhibitor [157]. These findings suggest that butyrate-producing capacity, rather than absolute bacterial abundance, may be critical for metabolic control during GDM.

Within Bacteroidetes, genera such as *Bacteroides* [89,90,136,137,140], *Prevotella* [92,95,146], and *Alistipes* [89,135,139] generally follow the phylum-level decline observed in GDM, whereas *Parabacteroides* often shows the opposite trend [61,104,135]. Notably, *Bacteroides* and *Parabacteroides* demonstrated strong predictive power for GDM in shotgun metagenomics analysis of 43 GDM and 81 healthy pregnancies [135]. High maternal Prevotellaceae abundance was associated with euglycemic pregnancies in a Czech cohort [92]. While Parabacteroides, typically enriched in overweight women, correlates positively with glucose tolerance [135,137], *Alistipes* shows inverse associations with hsCRP, fasting glucose, and OGTT results [26,89,135,137,139].

*Bifidobacterium*, which normally increases during healthy pregnancy, is notably reduced in GDM compared to normoglycaemic controls [89,104,135]. Metagenomic analysis of 27 GDM pregnancies identified Bifidobacterium as a potential protective factor, with its abundance rising after glycemic intervention, possibly enhancing SCFA production and improving glucose homeostasis [158,159]. Similarly, *Sutterella* (Proteobacteria) was more abundant in normoglycemic pregnancies than in GDM cases in a Czech cohort [92]. In T2D rat models, *Sutterella* improved glucose tolerance post-gastric bypass [160]. Interestingly, CRP—a well-established biomarker of systematic inflammation and proposed indicator for future onset of T2D—was also positively correlated with *Sutterella* during GDM progression [26], though this association is absent in healthy pregnancies, suggesting *Sutterella* may have distinct functional roles in different metabolic contexts.

### 7.4. Outstanding Questions: Methodological Variability and Transgenerational Effects?

Despite these observations, GDM-related microbial changes remain inconsistent across taxonomic levels, likely reflecting differences in gestational timing of sampling, cohort sample size, geographic locations, dietary patterns and sequencing methodologies. Further research is needed to clarify mechanistic pathways and establish causal relationships. Importantly, emerging evidence suggests that maternal gut dysbiosis may influence neonatal microbiota composition and immune development: reduced α-diversity and absence of unique taxa have been observed in newborns of GDM mothers compared to controls [46,161], raising questions about whether GDM-associated dysbiosis perpetuates metabolic dysfunction across generations through early-life microbial exposure.

**Table 3:** Gut microbial composition in gestational diabetes versus healthy pregnancies.

Year	Country	Sequencing	GDM (*n*)	Gestational Week	Healthy Ctrl (*n*)	Microbial Alterations
2012 [86]	Finland	16S rRNA (V1-V2)	15	13.84	76	↓: α diversity,
2017 [135]	China	Whole genome	43	21–29	81	≠: β diversity ↑: *Megamonas*, *Phascolarctobacterium*, *Parabacteroides* ↓: α diversity, *Clostridiales*, *Roseburia*, *Ruminiclostridium*, *Mitsukella*, *Corio-bacteriaceae*, *Eggerthella*, *Haemophilus*, *Aggregatibacter*, *Fusobacterium*
2017 [90]	Finland	16S rRNA	15	~12.9	60	↑: Clostridiales/Ruminococcaceae/unidentifies- genus and species
2018 [134]	China	16S rRNA (V3-V4)	26	Full term	23	≠: β diversity, ↓: *Faecalibacterium* Genara contributing to separation GDM+ and GMD-: *Lactobacillus*, *Faecalibacterium*, *Roseburia*, *Bacteroides*, *Prevotell*, *Porphyromonas*, *Fusobacterium*, *Sneathia*
2018 [136]	Brazil	16S rRNA (V4)	26	~33.89	42	≠: β diversity ↑: α diversity, F/B ratio, *Lachnosprira-ceae*, *Dorea*, *Subdoligranulum*, *Christen-senellaceae Ruminococcus*, *Collinsella* ↓: *Roseburia*, *Dialister*, *Akkermansia*
2018 [137]	Denmark	16S rRNA (V1-V2)	50	~28.4	157	↑: *Faecalibacterium*, *Blautia*, *Ruminococcus*, *Anaerotruncus*, *Leuconostoc*, *Granulicatella*, *Mogibacterium*, *Rothia*, *Collinsella*, *Actinomyces*, *Desulfovibrio* ↓: *Bacteroides*, *Sutterella*, *Ruminococcaceae*, *Erysipelotrichaceae*, *Veillonella*, *Marvinbryantia*, *Acetivibrio*, *Oscillibacter*, *Anaerosporobacter*, *Butyricicoccus*
2019 [138]	China	16S rRNA (V3-V4)	11	~31.2	11	↓: *Faecalibacterium* Increasing trend: Verrucomicrobia, *Roseburia*, *Lachnospiraceae*, *Blautia*
2019 [139]	China	Whole genome	23	~39.1	26	≠: β diversity ↑: *Bacteroides dorei*, *B.* sp. *3_1_3FAA* ↓: α diversity, *Lactobacillus casei*, *Alistipes putredinis*
2019 [140]	China	16S rRNA (V3-V4)	24 (successful control)	~26	16	↑: *Blautia*, *Eubacterium hallii*, *Subdoligranulum* ↓: *Faecalibacterium*
			12 (fail to control)	~25.2	16	≠: β diversity ↑: *Blautia*, *Eubacterium hallii* ↓: *Faecalibacterium*, *Subdoligranulum*
2020 [91]	Finland	Metage- nomics	53	~35.2	203	↑: *Megasphaera*
2020 [95]	China	16S rRNA (V3-V4)	31	~8	103	↓: *Copro-*, *Strepto-*, *Pepto-coccus*, *Prevotella*, *Intestinimonas*, *Veillonella*, *Desulfovibrio*
			31	~24	103	↑: *Holdemania*, *Megasphaera*, *Eggerthella* ↓: *Flavonifractor*, *Streptococcus*, *Coprococcus*
2020 [141]	China	16S rRNA (V3-V4)	30	~38.3	31	≠: β diversity ↑: *Hemophilus*, *γ-proteo-bacteria*
2020 [97]	China	16S rRNA (V3-V4)	45	24–28	45	≠: β diversity ↑: *Blautia*, *Faecalibacterium* ↓: α diversity, Bacteroidetes, *Christensenellaceae R7*, *Odoribacter*, *Butyricimonas*, *Akkermansia*
2021 [132]	China	16S rRNA (V3-V4)	23	~35.26	29	≠: β diversity ↑: α diversity (Shannon), F/B ratio, Firmicutes, *Dorea*, *Coprococcus*, *Ruminococcus*, *Blautia*, *Lachnospiraceae*, Clostridia, *Collinsella*, *Coriobacteriaceae* ↓: α diversity (Simpson), *Bacteroidetes*, Bacteroidales, Bacteroidia, *β-proteobacteria*, Alcalige-naceae, *Sutterella*, Burk-holderiales, *Pyramidobacter*, Dethiosulfovibrionacea
2021 [147]	Australia	16S rRNA (V6-V8)	29	~27.7	29	↑: *Blautia* ↓: α diversity, *Lachnospiraceae*
2021 [150]	Thailand	_ *	28 (successful control)	~24.75	38	↓: Lactobacillales
				~38.43	38	↑: F/B ratio
			13 (fail to control)	~25.23	38	↑: F/B ratio ↓: *Eubacteria*, Lactobacillales, *Bacteroidetes*, Enterobacteriaceae
				~38.23	38	↑: F/B ratio ↓: *Eubacteria*, Enterobacteriaceae
2022 [146]	Israel	16S rRNA (V4)	28	11–14	236	≠: β diversity ↓: *Ruminococcus*, *Lactobacillus*, Peptostreptococcaceae, *Megasphaera*, *Prevotella*, *Actinomyces*, *Collinsella*
2022 [92]	Czechia	16S rRNA (V3-V4)	29 (early onset)	T1	22	≠: β diversity ↓: Bacteroidia, *Prevotella*, *Sutterella*, *γ-proteobacteria*, *Fusobacteria*, *Methanobacteria*
			31 (late onset by FPG *)	T1	22	≠: β diversity ↑: *Enterococcus* ↓: α diversity, *Prevotella*, *Sutterella*, *Fusobacteria*
			22 (late onset by OGTT *)	T1	22	≠: β diversity ↑: *Erysipelotrichaceae UCG-003* ↓: α diversity, *Prevotella*, *Sutterella*, *Fusobacteria*
2023 [104]	China	16S rRNA (V4)	49	24–28	41	≠: β diversity ↑: α diversity, Firmicutes, *Roseburia*, *Lachnospira*, *Blautia*, *Parabacteroides* ↓: Actinobacteria, *Bifidobacterium*
			49	~39	39	≠: β diversity ↑: α diversity, Firmicutes, *Megamo-nas*, Lachnospiraceae, *Blautia*, *Para-bacteroides* ↓: Actinobacteria, *Bifidobacterium*
2023 [89]	China	Whole genome	120	~10.2	120	↓: α diversity Increasing trend: *Bacteroides massiliensis*, *E. coli*, *Fusobacterium mortiferum* Decreasing trend: *Ruminococcus bromii*, *Bifidobacterium dentium*
			120	~23.9	120	≠: β diversity Increasing trend: *B. massiliensis*, *Eubacterium ramulus*, *F. mortiferum* Decreasing trend: *CAG:58*, *R. bromii*, *Bacteroides ovatus*, *Alistipes putredinis*
			120	~33.6	120	≠: β diversity Increasing trend: *F. mortiferum* Decreasing trend: *R. bromii*, *B. dentium*, *B. ovatus*, *A. putredinis*
2024 [61]	Italy, Brazil, USA, China, South Africa, Austria	Varied	570	6–40	10378	≠: β diversity ↑: *Erysipelatoclostridium*, *Bacteroides*, *Parabacteroides*, *Alistipes* ↓: *Fusicatenibacter*, *Streptococcus*, *Coprococcus*, *Faecalibacterium*

* _: not mentioned, ≠: significantly different, ↑: increase, ↓: decrease, FPG: fasting plasma glucose, OGTT: oral glucose tolerance test.

## 8. Animal Models for Studying Gut Microbiota Composition During Pregnancy

Given the variability of microbial shifts across human studies due to experimental design and population characteristics, studies on controlled animal models have become essential, disentangling intrinsic mechanism of microbial changes and providing valuable complementary approaches to human studies for investigating gut microbiota dynamics during pregnancy (Table 4). However, such research remains relatively limited. Substantial differences also exist between human and animal microbial profiles due to variations in diet, physiology, and genetics [162]. Additionally, the much shorter gestational periods in rodents—19–21 days in mice and 22–23 days in rats compared to approximately 280 days in humans—pose challenges for translating findings to human conditions. Despite these limitations, animal models offer distinct advantages unavailable in human research. They enable rigorous control over diet and pregnancy induction, which is often constrained in human studies by ethical and practical considerations [162,163,164,165]. Furthermore, confounding variables such as lifestyle, medication, and environmental exposures can be standardised by controlling housing conditions and using uniform genetic backgrounds [162,163,164,165]. Critically, many tissues essential for microbial investigation during pregnancy such as segments of the gastrointestinal tract, placenta and maternal organs cannot be ethically collected from pregnant women, making animal models indispensable for mechanistic research.

### 8.1. Model Selection and Microbiota Comparability

Among available models, mice are the most widely used, either in direct studies or through human faecal microbiota transplantation experiments, to explore microbial composition and host–microbiota interactions. This preference reflects structural similarities between human and murine gut microbiota. Mouse gut bacteria is also dominated by four major phyla: Firmicutes (~55.8%), Bacteroidetes (~37.0%), Proteobacteria (~4.1%), and Actinobacteria (~2.0%) [166]. Several prevalent genera are also shared, including *Lactobacillus*, *Roseburia*, and *Ruminococcaceae UCG-014* within Firmicutes; *Bacteroides*, *Alloprevotella*, and *Alistipes* within Bacteroidetes; and *Escherichia-Shigella* within Proteobacteria [166]. Murine models have also provided substantial mechanistic insights into disease processes. For example, microbiota from individuals with IBD exacerbates colitis in mice [121], and gut dysbiosis has been implicated in type 1 diabetes, acute pancreatitis, and neurobehavioral disorders, mirroring patterns observed in humans [122,167,168,169,170]. Critically, as pregnancy progresses, microbial diversity patterns in mice closely parallel those observed in humans, characterised by minimal changes in α-diversity but significant shifts in β-diversity [94,171,172,173,174,175,176].

### 8.2. Phylum Level Changes: Consistent Bacteroidetes Reduction and Proteobacteria Enrichment

At the phylum level, murine pregnancy studies consistently report a decrease in Bacteroidetes accompanied by an increase in Proteobacteria [173,174,175,176,177,178]. Findings on the F/B ratio show greater variability across studies, though an overall upward trend is suggested, primarily driven by Bacteroidetes reduction [173,175,177]. Proteobacteria enrichment aligns with observations in healthy human pregnancies and has been associated with increased inflammation in late gestation [86,179]. Notably, mono-colonisation with *E. coli* (a Proteobacteria) in germ-free Swiss Webster mice promoted macrophage accumulation and disrupted glucose metabolism [86,179], suggesting potential metabolic consequences of *Proteobacteria* expansion during pregnancy.

### 8.3. Lower Taxonomic Rank Dynamics: From Nutrient Extractors to Cross-Species Conserved Taxa

Genus-level remodelling appears more consistent across murine studies compared to human studies. Many Firmicutes genera involved in nutrient extraction and energy metabolism, such as *Faecalibacterium*, *Roseburia*, *Allobaculum*, *Turicibacter*, *Ruminococcus*, *Streptococcus*, and *Lactococcus*, consistently increase during murine gestation. These taxa are recognised producers of short-chain fatty acids (SCFAs), glucose, and lactic acid, which play key critical roles in energy metabolism and immune homeostasis. Their enrichment may reflect adaptive responses supporting anti-inflammatory properties and immune tolerance during pregnancy [175,180].

### 8.4. Cross-Species Conserved Taxa: Biomarkers for Functional Investigation

Only a limited number of genera show similar enrichment patterns during late pregnancy in both humans and mice, notably *Streptococcus* (Firmicutes), *Bifidobacterium*, and *Collinsella* (Actinobacteria). In humans, *Bifidobacterium* and *Collinsella* are consistently enriched during late pregnancy, and comparable increases have been observed in mice at embryonic days 15–18 across strains including C57BL/6J, Swiss Webster, and BALB/c [94,171,180,181]. The enrichment of *Bifidobacterium* appears particularly protective in mice. Supplementation with specific strains has been shown to improve insulin sensitivity and reduce fat accumulation, partly through modulation of metabolic pathways in the gut and adipose tissue [182,183]. Oral administration of *Bifidobacterium* spp. also improved glucose tolerance by lowering inflammatory adipokines in both mice and rat L6 myoblast cells [184,185], suggesting conserved metabolic functions across rodent species and potential relevance to human pregnancy.

### 8.5. Rodent Specific Changes: Understanding Model Limitations and Opportunities

Other consistent changes in murine gut microbiota during pregnancy include increases in the opportunistic pathogen *Helicobacter* and the commensal genus *Mucispirillum*, which is abundant in rodents but much less common in humans [172,174,176,180,181]. The functional significance of *Mucispirillum* enrichment during pregnancy remains debated and is likely context dependent. In some contexts, *Mucispirillum* can antagonise *Salmonella* virulence and protect against colitis by limiting pathogen invasion [185]. Conversely, in Nod2/Cybb-deficient mice (Crohn’s disease-prone model), *Mucispirillum* exacerbated colitis, highlighting its pathobiont potential under dysbiotic or immune compromised conditions [186]. These species-specific patterns underscore the importance of interpreting murine findings within an appropriate biological context.

### 8.6. Translational Implications and Future Directions

Murine models remain among the most suitable systems for studying pregnancy-related microbiota dynamics, offering precise environmental control and access to tissues and mechanistic insights unavailable in human research. Specifically, microbial shifts consistently observed in mice that have not yet been systematically characterised in humans warrant targeted investigation in human cohorts. Conversely, taxa showing overlapping patterns in both species represent promising targets for mechanistic studies exploring their functional roles in supporting healthy pregnancy outcomes. Integration of human and murine data through carefully designed comparative studies may ultimately identify universal principles governing pregnancy-induced microbiota remodelling and reveal opportunities for microbiota-targeted interventions to prevent or mitigate pregnancy complications.

**Table 4:** Pregnancy-associated microbiota remodelling in murine models.

Year	Country	Sequencing	*n*	Gestational day	Compared to	Microbial Alterations
2015 [171]	Canada	16S rRNA (V3)	5 C57BL/6J mice	Post-conception	Pre-conception	≠: β diversity ↑: *Lactococcus*, *Turicibacter*, *Paenibacillus*, *Roseburia*, *Lachnobacterium*, *Allobaculum*, *Moryella*, *Bacteroides*, *Parabacteroides*, *Prevotella*, *Collinsella*, *Bifidobacterium*, *Bilophila*, *Escherichia*, *Akkermansia* ↓: Erysipelatotrichaceae, Clostridiaceae, *Anaerotruncus*, *Coprobacillus*, *Sarcina*
2016 [174]	Saudi Arabia	16S rRNA (V4)	6 Sprague- Dawley rat	20	6 Non-preg-nant	≠: β diversity ↑: α diversity, Proteobacteria, Clostridia-ceae, Erysipelotrichaceae, *Streptococcus*, *Clostridium* sp., *Turicibacter*, *Lactococcus*, *L. johnsonii*, Enterobacteriaceae, *Shigella*, *Helicobacter* ↓: Firmicutes, Bacteroidetes, Lactobacillaceae, Streptococcaceae, *Lactobacillus sp.*, *L. gallinarum*
2017 [172]	USA	16S rRNA (V4)	5 C57BL/6 mice	Late pregnancy	Early/ None	≠: β diversity ↑: Ruminococcaceae, Lachnospiraceae, *Rikenella*, *Helicobacter*, *Mucispirillum*, Desulfovibrionaceae, ↓: α diversity, *Bacteroides*, *S24-7*, *Prevotella*
2018 [180]	The Netherlands	Phylogenetic microarray (GI chip)	5 B6 mice	18	5 Non-pregnant	↓: α diversity
			5 BALB/c mice	18	5 Non-pregnant	↑: *Aerococcus*, *Allobaculum*, *Anaerotruncus*, *Butyrivibrio*, *Catenibacterium*, *Dialister*, *unclassified Clostridiales I*, *XIVa*, *XVI*, *C. herbivorans*, *C. perfringens*, *C. ramosum*, *Coprobacillus*, *Eubacterium*, *Lactococcus*, *Faecalibacterium*, *Roseburia*, *Lactobacillus*, *Ruminococcus*, *Solobacterium moorei*, *Staphylococcus*, *Streptococcus*, *Veilonella*, *Subdoligranulum*, *Bacteroides plebeius*, *Fibrobacter succinogenes*, *Porphyromonas asaccharolytica*, *Prevotella*, *unclassified Bacteroides*, *Acinetobacter*, *Atopobium*, *Collinsella*, *Corynebacterium*, *Eggerthella*, *Olsenella*, *Propionibacterium*, *Pasteurella*, *Pseudomonas*, *E. coli*, *Vibrio*, *Sphingomonas*, *Sutterella*, *Labrys methylaminiphilus*, *TM7*, *Acholeplasma*, *Fusobacterium*, *Helicobacter* ↓: α diversity, *C. lactifermentans*, *C. leptum*, *S. termitidis*, *Desulfovibrio*
2018 [187]	Canada	Targeted qPCR *	19–20 C57BL/ 6J mice	Full term	Pre-conception	↑: Bacteroidetes
2019 [177]	China	16S rRNA (V4)	12 MRL mice	Postnatal W4	12 Non-pregnant	↑: α diversity, Firmicutes, F/B ratio, Clostridiales, Lactobacillales, Verrucomicrobia, Acholeplasmatales ↓: Bacteroidetes, Bacteroidales, Erysi-pelotrichales, Desulfovibrionales
2019 [94]	Israel	16S rRNA (V4)	10 Swiss Webster mice	18	E0	≠: β diversity ↑: *S24-7*, *Bifidobacterium* ↓: *Coprococcus*, unclassified-Clostridiales, *Lactobacillus*, *Prevotella*, unclassified-Bacteroidales
2019 [181]	USA	16S rRNA (V4)	2 C57BL/6 (3–5 foetus per dam)	17–18, 19–20	E14–16	↑: *Allobaculum*, *Turicibacter*, *Mucispirillum* *schaedleri*, *S24-7*, *Bifidobacterium* ↓: *Lactobacillus*
2020 [176]	China	16S rRNA (V3-V4)	6 C57BL/6 mice	18	6 Pre-pregnant	≠: β diversity ↑: Proteobacteria, *Lactobacillus*, *S24-7*, *Allobaculum*, *Bacteroidales*, *Alloprevotella*, *Parasutterella*, Alcaligenaceae ↓: *Ruminococcaceae UCG_014*, *Akkermansia*
2020 [173]	The Netherlands	16S rRNA (V3-V4)	12 C57BL/6J-OlaHsd mice	18	Pre-pregnant	≠: β diversity ↑: F/B ratio, *Allobaculum stercoricanis*, *C. leptum* or *papyro-solvens*, *Faecalitalea cylindroides*, *Lactobacillus johnsonii*, *Roseburia faecis*, *Eubacteria plexicaudatum* ↓: *Barnesiella intestinihominis* or *viscericola*, *Porphyromonas pogonae*, *Alloprevotella rava*, *Odoribacter splanchnicus*, *Olsenella profuse*, *Parasutterella excrementihominis*
2023 [175]	Spain	16S rRNA (V3-V4)	9 C3H mice	12.5	9 Non-pregnant	↑: F/B ratio, *Faecalibacterium* ↓: Bacteroidetes, *Turicibacter*, *Anaerofustis*
2023 [178]	Japan	16S rRNA (V3-V4)	6 Avy × BALB/c mice	18	E11	↑: Actinobacteria, Proteobacteria ↓: Bacteroidetes

* Targeted qPCR: non-sequencing method using group or genus-specific primers, ≠: significantly different, ↑: increase, ↓: decrease, E: embryonic or gestational day, GI: gastrointestinal tract.

## 9. Discussion

**Figure 1:**
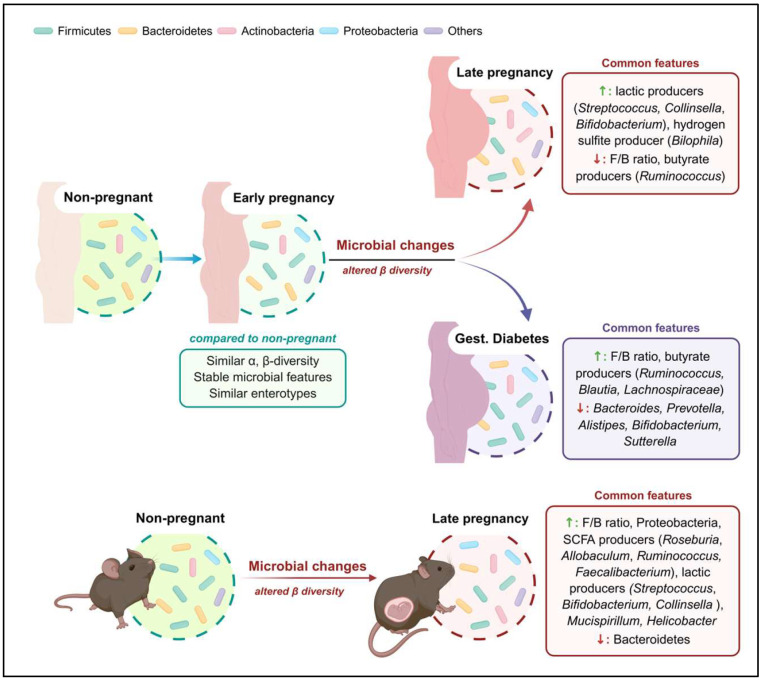
Gut microbiota remodelling during healthy pregnancy and gestational diabetes in human and murine models. Created in BioRender. Ly, D. (2026) https://BioRender.com/k19u658 (accessed on 18 February 2026).

Pregnancy is a physiological state characterised by profound metabolic and inflammatory adaptations which also bi-directionally influence gut microbiota composition [83] (Figure 1). During the first trimester, gut microbial composition remains largely comparable to that of non-pregnant individuals [25,61,86,92]. However, from the second trimester onwards, notable compositional shifts emerge, reflected by significant changes in β-diversity compared to the first trimester or non-pregnant controls [61,86,103]. At the phylum level, healthy pregnancy has often been associated with a decline in the F/B ratio, driven by reduced Firmicutes and increased Bacteroidetes abundance. These changes correlate with physiological gestational weight gain and elevated maternal fasting glucose levels in metabolically healthy pregnancies [62,106,112,113,114,115]. A distinctive hallmark of healthy pregnancy is increased *Actinobacteria* abundance, particularly its dominant genus *Bifidobacterium* [86,94,102]. *Bifidobacterium* appears especially beneficial during pregnancy, promoting placental morphogenesis, enhancing nutrient transport, and contributing to early neonatal immune development [106,107,108,109,110,111].

At the genus level, healthy pregnancy exhibits two contrasting patterns. First, *Ruminococcus* (a butyrate-producing genus) consistently declines during gestation [86,94,103]. This reduction may reflect selective suppression of certain *Ruminococcus* species that produce inflammatory polysaccharides activating immune responses and compromising gut barrier integrity, highlighting that not all butyrate producers are uniformly beneficial. Conversely, the enrichment of lactic acid-producing genera, such as *Bifidobacterium*, *Collinsella*, and *Streptococcus,* is frequently reported [61,86,95,103]. These taxa may enhance nutrient bioavailability and promote a healthier gut environment; notably, *Streptococcus* and *Bifidobacterium* have been associated with reduced risk of preterm birth [127,131]. However, the precise mechanistic roles of these taxa in maintaining healthy pregnancy outcomes remain controversial.

GDM is the most common metabolic disorder during pregnancy. The gut microbiota in GDM exhibits alterations that partially resemble those in T2D [137]. A defining feature is the elevated F/B ratio [26,95,132,136,150], similar to T2D patterns but opposite to healthy pregnancy [89,91,102,104], suggesting pathological rather than physiological remodelling. Importantly, *Bifidobacterium*—a beneficial strain increased in healthy pregnancies and associated with placental morphogenesis and nutrient bioavailability [94,106,107]—is consistently reduced in GDM [26,61,95,104]. Additional GDM-associated changes include significant β-diversity shifts [61,89,92,97,104,134,135,139,140,141,146], increased *Ruminococcus* abundance [90,95,132,136,137] and the reduced levels of Bacteroidetes genera [97,104,132,134,136], *Clostridium*, and *Akkermansia* compared to normoglycemic pregnancies [26,61,95,104,188]. These microbial patterns closely mirror T2D dysbiosis, suggesting shared pathological mechanisms.

Despite these similarities, important distinctions exist between GDM and T2D dysbiosis that suggest pregnancy-specific metabolic challenges. While butyrate-producing bacteria typically decline in T2D, they remain unchanged (*Faecalibacterium*, *Roseburia*) or even increased (*Blautia*, *Ruminococcus*, *Lachnospiraceae*) in GDM [95,97,104,132,136,137,138,140,147]. This divergence indicates a more complex functional role for butyrate producers in pregnancy, potentially driven by factors beyond glucose homeostasis, such as foetal nutrient demands, placental development, or pregnancy-specific immune tolerance mechanisms. These findings underscore the need for pregnancy-specific mechanistic research rather than direct extrapolation from T2D models. Clarifying the underlying mechanisms driving GDM-associated dysbiosis remains critical for developing targeted interventions.

Gut microbiota changes during pregnancy have also been investigated in murine models, which offer value for mechanistic investigation due to similarities between mice and humans in digestive anatomy and predominant gut microbial composition. However, existing murine pregnancy microbiota studies remain limited in number and present conflicting observations. Notably, enrichment of two *Actinobacteria* genera (*Bifidobacterium* and *Collinsella*) have been consistently observed during late pregnancy in both humans and mice [94,171,180,181]. This shared feature underscores the potential biological significance of Proteobacteria and Actinobacteria in pregnancy-related gut microbiota changes across species. Conversely, divergent patterns have been reported for the two predominant phyla, Firmicutes and Bacteroidetes, between pregnant mice and humans, highlighting the importance of cautious interpretation when extrapolating findings from animal models to human physiology. Such inconsistencies may reflect species-specific differences in microbial functional capacity, baseline microbiota composition, or fundamental physiological differences in human versus murine pregnancy (e.g., gestation duration, placental architecture, immune development timelines).

Although the gut microbiota represents a vast and largely invisible ecosystem, it remains a modifiable target and has a well-established association with both healthy pregnancy and GDM pathogenesis. This recognition opens clinical opportunities for microbiota-informed interventions. Clinical strategies should emphasise personalised dietary guidance informed by individual microbiota composition, identification of beneficial versus potentially pathogenic microbial taxa, and targeted supplementation approaches. Emerging therapeutic options include faecal microbiota transplantation, prebiotics, probiotics, synbiotics, and postbiotics—each offering distinct mechanistic advantages. Prebiotics enhance growth of endogenous beneficial bacteria; probiotics and synbiotics introduce or reinforce beneficial taxa; postbiotics provide metabolic byproducts of fermentation without requiring viable organisms. These microbiota-modulating interventions represent promising, practical avenues for mitigating GDM risk, optimising metabolic adaptations during healthy pregnancy, and ultimately supporting maternal and foetal health throughout gestation and beyond.

## 10. Limitations

This narrative review synthesising gut microbiota studies during healthy pregnancy and gestational diabetes across human and mouse models faces several important limitations. Methodological heterogeneity including variable sequencing technologies (16S rRNA, shotgun metagenomics, genus-specific probes) and inconsistent taxonomic reporting limits cross-study comparability and quantitative synthesis. Population heterogeneity further complicates interpretation, with variations in gestational age, maternal metabolic state, GDM diagnostic criteria, and inconsistent adjustment for confounders (BMI, diet, medications, parity) potentially complicating conclusion strength. The gut microbiota represents a highly interconnected system, yet individual studies typically examine only specific bacterial taxa or functional domains, risking speculative interpretations when direct evidence is insufficient. Additionally, cross-study comparisons may reflect contextual differences (diet, lifestyle, geography, environmental exposures) rather than true pregnancy related microbial signatures. Despite these limitations, this comprehensive synthesis advances the field by comprehensively integrating findings across diverse experimental models and populations, thereby establishing a robust evidence base that identifies critical knowledge gaps, prioritises key research directions, and provides a platform for hypothesis-driven investigations to clarify pregnancy-associated microbiota dynamics and their metabolic consequences.

## 11. Conclusions

Pregnancy induces profound metabolic and immunological changes that shape gut microbiota composition. Evidence consistently highlights increased Actinobacteria, particularly Bifidobacterium, as a hallmark of healthy pregnancy, while shifts in Firmicutes and Bacteroidetes remain variable. In contrast, GDM exhibits microbial patterns resembling T2D, including elevated F/B ratios and reduced *Bifidobacterium*, suggesting pathological rather than physiological adaptations. However, inconsistencies across studies driven by differences in geography, methodology, and gestational timing underscore the need for standardised approaches and longitudinal designs.

Animal models provide mechanistic insights but require cautious interpretation due to species-specific differences. Future research should prioritise functional studies linking microbial shifts to metabolic pathways, explore microbiome-based interventions, and assess their impact on maternal and neonatal outcomes. Ultimately, integrating microbiome-informed strategies into clinical care through diet and gut-targeted therapies offers promising avenues for improving pregnancy health and reducing GDM risk.

## Data Availability

No new data was created.

## Abbreviations

The following abbreviations are used in this manuscript:

GDM	Gestational diabetes mellitus
GI	Gastrointestinal
T2D	Type 2 diabetes
SCFA	Short chain fatty acid
IBD	Inflammatory bowel disease
BMI	Body mass index
F/B	Firmicutes-to-Bacteroidetes
FPG	Fasting plasma glucose
OGTT	Oral glucose tolerance test
HbA1c	Haemoglobin A1c
HOMA-IR	Homeostatic model assessment of insulin resistance
HDL	High-density lipoprotein
qPCR	Quantitative Polymerase Chain Reaction

## References

[1] Srivastava A., Prabhakar M.R., Mohanty A., Meena S.S. (2022). Influence of gut microbiome on the human physiology. Syst. Microbiol. Biomanuf..

[2] De Santis S., Cavalcanti E., Mastronardi M., Jirillo E., Chieppa M. (2015). Nutritional keys for intestinal barrier modulation. Front. Immunol..

[3] Thursby E., Juge N. (2017). Introduction to the human gut microbiota. Biochem. J..

[4] Gill S.R., Pop M., DeBoy R.T., Eckburg P.B., Turnbaugh P.J., Samuel B.S., Gordon J.I., Relman D.A., Fraser-Liggett C.M., Nelson K.E. (2006). Metagenomic analysis of the human distal gut microbiome. Science.

[5] Jandhyala S.M., Talukdar R., Subramanyam C., Vuyyuru H., Sasikala M., Reddy D.N. (2015). Role of the normal gut microbiota. World J. Gastroenterol. WJG.

[6] Adak A., Khan M.R. (2019). An insight into gut microbiota and its functionalities. Cell. Mol. Life Sci..

[7] Backhed F., Ley R.E., Sonnenburg J.L., Peterson D.A., Gordon J.I. (2005). Host-bacterial mutualism in the human intestine. Science.

[8] Rinninella E., Raoul P., Cintoni M., Franceschi F., Miggiano G.A.D., Gasbarrini A., Mele M.C. (2019). What is the healthy gut microbiota composition? A changing ecosystem across age, environment, diet, and diseases. Microorganisms.

[9] Collado M.C., Devkota S., Ghosh T.S. (2024). Gut microbiome: A biomedical revolution. Nat. Rev. Gastroenterol. Hepatol..

[10] Korpela K., de Vos W.M. (2022). Infant gut microbiota restoration: State of the art. Gut Microbes.

[11] Shenhav L., Fehr K., Reyna M.E., Petersen C., Dai D.L., Dai R., Breton V., Rossi L., Smieja M., Simons E. (2024). Microbial colonization programs are structured by breastfeeding and guide healthy respiratory development. Cell.

[12] Kostic A.D., Gevers D., Siljander H., Vatanen T., Hyötyläinen T., Hämäläinen A.-M., Peet A., Tillmann V., Pöhö P., Mattila I. (2015). The dynamics of the human infant gut microbiome in development and in progression toward type 1 diabetes. Cell Host Microbe.

[13] Korpela K., Zijlmans M., Kuitunen M., Kukkonen K., Savilahti E., Salonen A., De Weerth C., De Vos W. (2017). Childhood BMI in relation to microbiota in infancy and lifetime antibiotic use. Microbiome.

[14] Abrahamsson T.R., Jakobsson H.E., Andersson A.F., Björkstén B., Engstrand L., Jenmalm M.C. (2012). Low diversity of the gut microbiota in infants with atopic eczema. J. Allergy Clin. Immunol..

[15] Abrahamsson T.R., Jakobsson H.E., Andersson A.F., Björkstén B., Engstrand L., Jenmalm M.C. (2014). Low gut microbiota diversity in early infancy precedes asthma at school age. Clin. Exp. Allergy.

[16] Azad M.B., Konya T., Guttman D.S., Field C., Sears M., HayGlass K., Mandhane P., Turvey S., Subbarao P., Becker A. (2015). Infant gut microbiota and food sensitization: Associations in the first year of life. Clin. Exp. Allergy.

[17] Zuffa S., Schimmel P., Gonzalez-Santana A., Belzer C., Knol J., Bölte S., Falck-Ytter T., Forssberg H., Swann J., Diaz Heijtz R. (2023). Early-life differences in the gut microbiota composition and functionality of infants at elevated likelihood of developing autism spectrum disorder. Transl. Psychiatry.

[18] Federation I.D. Facts & Figures. https://idf.org/about-diabetes/diabetes-facts-figures/.

[19] Lee C.J., Bergman B.K., Gou R., Williamson S., Boye K.S. (2025). Prevalence, demographic and clinical characteristics of individuals with early onset type 2 diabetes in the USA: An Nhanes analysis 1999–2020. Diabetes Ther..

[20] Sweeting A., Hannah W., Backman H., Catalano P., Feghali M., Herman W.H., Hivert M.-F., Immanuel J., Meek C., Oppermann M.L. (2024). Epidemiology and management of gestational diabetes. Lancet.

[21] Quadir H. (2021). Current therapeutic use of metformin during pregnancy: Maternal changes, postnatal effects and safety. Cureus.

[22] Organization of Teratology Information Specialists (OTIS) (2023). Gestational Diabetes Mother to Baby|Fact Sheets.

[23] Kc K., Shakya S., Zhang H. (2015). Gestational diabetes mellitus and macrosomia: A literature review. Ann. Nutr. Metab..

[24] Hu P., Chen X., Chu X., Fan M., Ye Y., Wang Y., Han M., Yang X., Yuan J., Zha L. (2021). Association of gut microbiota during early pregnancy with risk of incident gestational diabetes mellitus. J. Clin. Endocrinol. Metab..

[25] Ma S., You Y., Huang L., Long S., Zhang J., Guo C., Zhang N., Wu X., Xiao Y., Tan H. (2020). Alterations in gut microbiota of gestational diabetes patients during the first trimester of pregnancy. Front. Cell. Infect. Microbiol..

[26] Ferrocino I., Ponzo V., Gambino R., Zarovska A., Leone F., Monzeglio C., Goitre I., Rosato R., Romano A., Grassi G. (2018). Changes in the gut microbiota composition during pregnancy in patients with gestational diabetes mellitus (GDM). Sci. Rep..

[27] Sarkar A., Yoo J.Y., Valeria Ozorio Dutra S., Morgan K.H., Groer M. (2021). The association between early-life gut microbiota and long-term health and diseases. J. Clin. Med..

[28] Tao Y.-W., Gu Y.-L., Mao X.-Q., Zhang L., Pei Y.-F. (2020). Effects of probiotics on type II diabetes mellitus: A meta-analysis. J. Transl. Med..

[29] Memon H., Abdulla F., Reljic T., Alnuaimi S., Serdarevic F., Asimi Z.V., Kumar A., Semiz S. (2023). Effects of combined treatment of probiotics and metformin in management of type 2 diabetes: A systematic review and meta-analysis. Diabetes Res. Clin. Pract..

[30] Vrieze A., Van Nood E., Holleman F., Salojärvi J., Kootte R.S., Bartelsman J.F., Dallinga–Thie G.M., Ackermans M.T., Serlie M.J., Oozeer R. (2012). Transfer of intestinal microbiota from lean donors increases insulin sensitivity in individuals with metabolic syndrome. Gastroenterology.

[31] Kootte R.S., Levin E., Salojärvi J., Smits L.P., Hartstra A.V., Udayappan S.D., Hermes G., Bouter K.E., Koopen A.M., Holst J.J. (2017). Improvement of insulin sensitivity after lean donor feces in metabolic syndrome is driven by baseline intestinal microbiota composition. Cell Metab..

[32] Wahlström A., Brumbaugh A., Sjöland W., Olsson L., Wu H., Henricsson M., Lundqvist A., Makki K., Hazen S.L., Bergström G. (2024). Production of deoxycholic acid by low-abundant microbial species is associated with impaired glucose metabolism. Nat. Commun..

[33] Bäckhed F., Ding H., Wang T., Hooper L.V., Koh G.Y., Nagy A., Semenkovich C.F., Gordon J.I. (2004). The gut microbiota as an environmental factor that regulates fat storage. Proc. Natl. Acad. Sci. USA.

[34] Sender R., Fuchs S., Milo R. (2016). Revised Estimates for the Number of Human and Bacteria Cells in the Body. PLoS Biol..

[35] Ursell L.K., Haiser H.J., Van Treuren W., Garg N., Reddivari L., Vanamala J., Dorrestein P.C., Turnbaugh P.J., Knight R. (2014). The intestinal metabolome: An intersection between microbiota and host. Gastroenterology.

[36] Qin J., Li R., Raes J., Arumugam M., Burgdorf K.S., Manichanh C., Nielsen T., Pons N., Levenez F., Yamada T. (2010). A human gut microbial gene catalogue established by metagenomic sequencing. Nature.

[37] Ventura M., O’flaherty S., Claesson M.J., Turroni F., Klaenhammer T.R., Van Sinderen D., O’toole P.W. (2009). Genome-scale analyses of health-promoting bacteria: Probiogenomics. Nat. Rev. Microbiol..

[38] Milani C., Duranti S., Bottacini F., Casey E., Turroni F., Mahony J., Belzer C., Delgado Palacio S., Arboleya Montes S., Mancabelli L. (2017). The first microbial colonizers of the human gut: Composition, activities, and health implications of the infant gut microbiota. Microbiol. Mol. Biol. Rev..

[39] Hugon P., Dufour J.-C., Colson P., Fournier P.-E., Sallah K., Raoult D. (2015). A comprehensive repertoire of prokaryotic species identified in human beings. Lancet Infect. Dis..

[40] Li J., Jia H., Cai X., Zhong H., Feng Q., Sunagawa S., Arumugam M., Kultima J.R., Prifti E., Nielsen T. (2014). An integrated catalog of reference genes in the human gut microbiome. Nat. Biotechnol..

[41] King C.H., Desai H., Sylvetsky A.C., LoTempio J., Ayanyan S., Carrie J., Crandall K.A., Fochtman B.C., Gasparyan L., Gulzar N. (2019). Baseline human gut microbiota profile in healthy people and standard reporting template. PLoS ONE.

[42] Rajilić-Stojanović M., Smidt H., De Vos W.M. (2007). Diversity of the human gastrointestinal tract microbiota revisited. Environ. Microbiol..

[43] Kovatcheva-Datchary P., Tremaroli V., Bäckhed F., Rosenberg E., DeLong E.F., Lory S., Stackebrandt E., Thompson F. (2013). The Gut Microbiota. The Prokaryotes: Human Microbiology.

[44] Arumugam M., Raes J., Pelletier E., Le Paslier D., Yamada T., Mende D.R., Fernandes G.R., Tap J., Bruls T., Batto J.-M. (2011). Enterotypes of the human gut microbiome. Nature.

[45] Collado M.C., Rautava S., Aakko J., Isolauri E., Salminen S. (2016). Human gut colonisation may be initiated in utero by distinct microbial communities in the placenta and amniotic fluid. Sci. Rep..

[46] Hu J., Nomura Y., Bashir A., Fernandez-Hernandez H., Itzkowitz S., Pei Z., Stone J., Loudon H., Peter I. (2013). Diversified microbiota of meconium is affected by maternal diabetes status. PLoS ONE.

[47] Moles L., Gomez M., Heilig H., Bustos G., Fuentes S., de Vos W., Fernandez L., Rodríguez J.M., Jimenez E. (2013). Bacterial diversity in meconium of preterm neonates and evolution of their fecal microbiota during the first month of life. PLoS ONE.

[48] Ardissone A.N., de la Cruz D.M., Davis-Richardson A.G., Rechcigl K.T., Li N., Drew J.C., Murgas-Torrazza R., Sharma R., Hudak M.L., Triplett E.W. (2014). Meconium microbiome analysis identifies bacteria correlated with premature birth. PLoS ONE.

[49] Hansen R., Scott K.P., Khan S., Martin J.C., Berry S.H., Stevenson M., Okpapi A., Munro M.J., Hold G.L. (2015). First-pass meconium samples from healthy term vaginally-delivered neonates: An analysis of the microbiota. PLoS ONE.

[50] Urushiyama D., Suda W., Ohnishi E., Araki R., Kiyoshima C., Kurakazu M., Sanui A., Yotsumoto F., Murata M., Nabeshima K. (2017). Microbiome profile of the amniotic fluid as a predictive biomarker of perinatal outcome. Sci. Rep..

[51] Stinson L.F., Boyce M.C., Payne M.S., Keelan J.A. (2019). The not-so-sterile womb: Evidence that the human fetus is exposed to bacteria prior to birth. Front. Microbiol..

[52] Aagaard K., Ma J., Antony K.M., Ganu R., Petrosino J., Versalovic J. (2014). The placenta harbors a unique microbiome. Sci. Transl. Med..

[53] Jiménez E., Fernández L., Marín M.L., Martín R., Odriozola J.M., Nueno-Palop C., Narbad A., Olivares M., Xaus J., Rodríguez J.M. (2005). Isolation of commensal bacteria from umbilical cord blood of healthy neonates born by cesarean section. Curr. Microbiol..

[54] Yassour M., Vatanen T., Siljander H., Hämäläinen A.-M., Härkönen T., Ryhänen S.J., Franzosa E.A., Vlamakis H., Huttenhower C., Gevers D. (2016). Natural history of the infant gut microbiome and impact of antibiotic treatment on bacterial strain diversity and stability. Sci. Transl. Med..

[55] Dominguez-Bello M.G., Costello E.K., Contreras M., Magris M., Hidalgo G., Fierer N., Knight R. (2010). Delivery mode shapes the acquisition and structure of the initial microbiota across multiple body habitats in newborns. Proc. Natl. Acad. Sci. USA.

[56] Jakobsson H.E., Abrahamsson T.R., Jenmalm M.C., Harris K., Quince C., Jernberg C., Björkstén B., Engstrand L., Andersson A.F. (2014). Decreased gut microbiota diversity, delayed Bacteroidetes colonisation and reduced Th1 responses in infants delivered by caesarean section. Gut.

[57] Salminen S., Gibson G.R., McCartney A.L., Isolauri E. (2004). Influence of mode of delivery on gut microbiota composition in seven year old children. Gut.

[58] Bäckhed F., Fraser C.M., Ringel Y., Sanders M.E., Sartor R.B., Sherman P.M., Versalovic J., Young V., Finlay B.B. (2012). Defining a healthy human gut microbiome: Current concepts, future directions, and clinical applications. Cell Host Microbe.

[59] Van Hul M., Cani P.D., Petitfils C., De Vos W.M., Tilg H., El-Omar E.M. (2024). What defines a healthy gut microbiome?. Gut.

[60] Human Microbiome Project Consortium (2012). Structure, function and diversity of the healthy human microbiome. Nature.

[61] Xiao L., Zhou T., Zuo Z., Sun N., Zhao F. (2024). Spatiotemporal patterns of the pregnancy microbiome and links to reproductive disorders. Sci. Bull..

[62] Turnbaugh P.J., Hamady M., Yatsunenko T., Cantarel B.L., Duncan A., Ley R.E., Sogin M.L., Jones W.J., Roe B.A., Affourtit J.P. (2009). A core gut microbiome in obese and lean twins. Nature.

[63] Eckburg P.B., Bik E.M., Bernstein C.N., Purdom E., Dethlefsen L., Sargent M., Gill S.R., Nelson K.E., Relman D.A. (2005). Diversity of the human intestinal microbial flora. Science.

[64] Tap J., Mondot S., Levenez F., Pelletier E., Caron C., Furet J.P., Ugarte E., Muñoz-Tamayo R., Paslier D.L., Nalin R. (2009). Towards the human intestinal microbiota phylogenetic core. Environ. Microbiol..

[65] Walters W.A., Xu Z., Knight R. (2014). Meta-analyses of human gut microbes associated with obesity and IBD. FEBS Lett..

[66] Seo H., Yoon S.Y., Ul-Haq A., Jo S., Kim S., Rahim M.A., Park H.-A., Ghorbanian F., Kim M.J., Lee M.-Y. (2023). The effects of iron deficiency on the gut microbiota in women of childbearing age. Nutrients.

[67] Huang T., Liang X., Bao H., Ma G., Tang X., Luo H., Xiao X. (2024). Multi-omics analysis reveals the associations between altered gut microbiota, metabolites, and cytokines during pregnancy. Msystems.

[68] Costea P.I., Hildebrand F., Arumugam M., Bäckhed F., Blaser M.J., Bushman F.D., De Vos W.M., Ehrlich S.D., Fraser C.M., Hattori M. (2018). Enterotypes in the landscape of gut microbial community composition. Nat. Microbiol..

[69] Falony G., Joossens M., Vieira-Silva S., Wang J., Darzi Y., Faust K., Kurilshikov A., Bonder M.J., Valles-Colomer M., Vandeputte D. (2016). Population-level analysis of gut microbiome variation. Science.

[70] Zupancic M.L., Cantarel B.L., Liu Z., Drabek E.F., Ryan K.A., Cirimotich S., Jones C., Knight R., Walters W.A., Knights D. (2012). Analysis of the gut microbiota in the old order Amish and its relation to the metabolic syndrome. PLoS ONE.

[71] Wu G.D., Chen J., Hoffmann C., Bittinger K., Chen Y.-Y., Keilbaugh S.A., Bewtra M., Knights D., Walters W.A., Knight R. (2011). Linking long-term dietary patterns with gut microbial enterotypes. Science.

[72] Ringel Y., Maharshak N., Ringel-Kulka T., Wolber E.A., Sartor R.B., Carroll I.M. (2015). High throughput sequencing reveals distinct microbial populations within the mucosal and luminal niches in healthy individuals. Gut Microbes.

[73] Hayashi H., Takahashi R., Nishi T., Sakamoto M., Benno Y. (2005). Molecular analysis of jejunal, ileal, caecal and recto-sigmoidal human colonic microbiota using 16S rRNA gene libraries and terminal restriction fragment length polymorphism. J. Med. Microbiol..

[74] Odamaki T., Kato K., Sugahara H., Hashikura N., Takahashi S., Xiao J.-Z., Abe F., Osawa R. (2016). Age-related changes in gut microbiota composition from newborn to centenarian: A cross-sectional study. BMC Microbiol..

[75] Ross F.C., Patangia D., Grimaud G., Lavelle A., Dempsey E.M., Ross R.P., Stanton C. (2024). The interplay between diet and the gut microbiome: Implications for health and disease. Nat. Rev. Microbiol..

[76] Nagpal R., Tsuji H., Takahashi T., Nomoto K., Kawashima K., Nagata S., Yamashiro Y. (2017). Ontogenesis of the gut microbiota composition in healthy, full-term, vaginally born and breast-fed infants over the first 3 years of life: A quantitative bird’s-eye view. Front. Microbiol..

[77] Yatsunenko T., Rey F.E., Manary M.J., Trehan I., Dominguez-Bello M.G., Contreras M., Magris M., Hidalgo G., Baldassano R.N., Anokhin A.P. (2012). Human gut microbiome viewed across age and geography. Nature.

[78] De la Fuente M., Franchi L., Araya D., Díaz-Jiménez D., Olivares M., Álvarez-Lobos M., Golenbock D., González M.-J., López-Kostner F., Quera R. (2014). Escherichia coli isolates from inflammatory bowel diseases patients survive in macrophages and activate NLRP3 inflammasome. Int. J. Med. Microbiol..

[79] Gevers D., Kugathasan S., Denson L.A., Vázquez-Baeza Y., Van Treuren W., Ren B., Schwager E., Knights D., Song S.J., Yassour M. (2014). The treatment-naive microbiome in new-onset Crohn’s disease. Cell Host Microbe.

[80] Hill D.A., Siracusa M.C., Abt M.C., Kim B.S., Kobuley D., Kubo M., Kambayashi T., LaRosa D.F., Renner E.D., Orange J.S. (2012). Commensal bacteria–derived signals regulate basophil hematopoiesis and allergic inflammation. Nat. Med..

[81] Patterson E., Ryan P.M., Cryan J.F., Dinan T.G., Ross R.P., Fitzgerald G.F., Stanton C. (2016). Gut microbiota, obesity and diabetes. Postgrad. Med. J..

[82] Pitocco D., Di Leo M., Tartaglione L., De Leva F., Petruzziello C., Saviano A., Pontecorvi A., Ojetti V. (2020). The role of gut microbiota in mediating obesity and diabetes mellitus. Eur. Rev. Med. Pharmacol. Sci..

[83] Kepley J.M., Bates K., Mohiuddin S.S. (2023). Physiology, maternal changes. StatPearls [Internet].

[84] Weng J., Couture C., Girard S. (2023). Innate and adaptive immune systems in physiological and pathological pregnancy. Biology.

[85] Barbour L.A., McCurdy C.E., Hernandez T.L., Kirwan J.P., Catalano P.M., Friedman J.E. (2007). Cellular mechanisms for insulin resistance in normal pregnancy and gestational diabetes. Diabetes Care.

[86] Koren O., Goodrich J.K., Cullender T.C., Spor A., Laitinen K., Bäckhed H.K., Gonzalez A., Werner J.J., Angenent L.T., Knight R. (2012). Host remodeling of the gut microbiome and metabolic changes during pregnancy. Cell.

[87] Gomez-Arango L.F., Barrett H.L., McIntyre H.D., Callaway L.K., Morrison M., Dekker Nitert M. (2016). Connections between the gut microbiome and metabolic hormones in early pregnancy in overweight and obese women. Diabetes.

[88] Vazquez-Moreno M., Perez-Herrera A., Locia-Morales D., Dizzel S., Meyre D., Stearns J.C., Cruz M. (2021). Association of gut microbiome with fasting triglycerides, fasting insulin and obesity status in Mexican children. Pediatr. Obes..

[89] Sun Z., Pan X.F., Li X., Jiang L., Hu P., Wang Y., Ye Y., Wu P., Zhao B., Xu J. (2023). The Gut Microbiome Dynamically Associates with Host Glucose Metabolism throughout Pregnancy: Longitudinal Findings from a Matched Case-Control Study of Gestational Diabetes Mellitus. Adv. Sci..

[90] Mokkala K., Houttu N., Vahlberg T., Munukka E., Rönnemaa T., Laitinen K. (2017). Gut microbiota aberrations precede diagnosis of gestational diabetes mellitus. Acta Diabetol..

[91] Mokkala K., Paulin N., Houttu N., Koivuniemi E., Pellonperä O., Khan S., Pietilä S., Tertti K., Elo L.L., Laitinen K. (2021). Metagenomics analysis of gut microbiota in response to diet intervention and gestational diabetes in overweight and obese women: A randomised, double-blind, placebo-controlled clinical trial. Gut.

[92] Vavreckova M., Galanova N., Kostovcik M., Krystynik O., Ivanovova E., Roubalova R., Jiraskova Zakostelska Z., Friedecky D., Friedecka J., Haluzik M. (2022). Specific gut bacterial and fungal microbiota pattern in the first half of pregnancy is linked to the development of gestational diabetes mellitus in the cohort including obese women. Front. Endocrinol..

[93] Avershina E., Storrø O., Øien T., Johnsen R., Wilson R., Egeland T., Rudi K. (2013). Bifidobacterial succession and correlation networks in a large unselected cohort of mothers and their children. Appl. Environ. Microbiol..

[94] Nuriel-Ohayon M., Neuman H., Ziv O., Belogolovski A., Barsheshet Y., Bloch N., Uzan A., Lahav R., Peretz A., Frishman S. (2019). Progesterone increases Bifidobacterium relative abundance during late pregnancy. Cell Rep..

[95] Zheng W., Xu Q., Huang W., Yan Q., Chen Y., Zhang L., Tian Z., Liu T., Yuan X., Liu C. (2020). Gestational diabetes mellitus is associated with reduced dynamics of gut microbiota during the first half of pregnancy. MSystems.

[96] Stevens A.J., Heiwari T.M., Rich F.J., Bradley H.A., Gur T.L., Galley J.D., Kennedy M.A., Dixon L.A., Mulder R.T., Rucklidge J.J. (2024). Randomised control trial indicates micronutrient supplementation may support a more robust maternal microbiome for women with antenatal depression during pregnancy. Clin. Nutr..

[97] Liu Y., Qin S., Feng Y., Song Y., Lv N., Liu F., Zhang X., Wang S., Wei Y., Li S. (2020). Perturbations of gut microbiota in gestational diabetes mellitus patients induce hyperglycemia in germ-free mice. J. Dev. Orig. Health Dis..

[98] Abdullah B., Idorus M.Y., Daud S., Aazmi S., Pillai T.K., Zain Z.M. (2023). Gut Microbiota Composition in the First and Third Trimester of Pregnancy among Malay Women is Associated with Body Mass Index: A Pilot Study. Malays. J. Med. Sci. MJMS.

[99] Butte N.F. (2000). Carbohydrate and lipid metabolism in pregnancy: Normal compared with gestational diabetes mellitus. Am. J. Clin. Nutr..

[100] Yang T., Santisteban M.M., Rodriguez V., Li E., Ahmari N., Carvajal J.M., Zadeh M., Gong M., Qi Y., Zubcevic J. (2015). Gut dysbiosis is linked to hypertension. hypertension.

[101] Liu X., Zhang F., Wang Z., Zhang T., Teng C., Wang Z. (2021). Altered gut microbiome accompanying with placenta barrier dysfunction programs pregnant complications in mice caused by graphene oxide. Ecotoxicol. Environ. Saf..

[102] Gao Y., Zhang J., Chen H., Jin X., Lin Z., Fan C., Shan Z., Teng W., Li J. (2024). Dynamic changes in the gut microbiota during three consecutive trimesters of pregnancy and their correlation with abnormal glucose and lipid metabolism. Eur. J. Med. Res..

[103] Chen X., Wu R., Li L., Zeng Y., Chen J., Wei M., Feng Y., Chen G., Wang Y., Lin L. (2023). Pregnancy-induced changes to the gut microbiota drive macrophage pyroptosis and exacerbate septic inflammation. Immunity.

[104] Liu N., Sun Y., Wang Y., Ma L., Zhang S., Lin H. (2023). Composition of the intestinal microbiota and its variations between the second and third trimesters in women with gestational diabetes mellitus and without gestational diabetes mellitus. Front. Endocrinol..

[105] Li Z., Zhang Y., Wang L., Deng T.K., Chiu W.-H., Ming W.-K., Xu C., Xiao X. (2024). Microbiota of pregnancy, placenta and newborns in the third trimester: A randomized controlled study. Heliyon.

[106] Santacruz A., Collado M.C., Garcia-Valdes L., Segura M., Martín-Lagos J., Anjos T., Martí-Romero M., Lopez R., Florido J., Campoy C. (2010). Gut microbiota composition is associated with body weight, weight gain and biochemical parameters in pregnant women. Br. J. Nutr..

[107] Lopez-Tello J., Schofield Z., Kiu R., Dalby M.J., van Sinderen D., Le Gall G., Sferruzzi-Perri A.N., Hall L.J. (2022). Maternal gut microbiota Bifidobacterium promotes placental morphogenesis, nutrient transport and fetal growth in mice. Cell. Mol. Life Sci..

[108] Tannock G.W. (2010). Analysis of bifidobacterial populations in bowel ecology studies. Bifidobacteria: Genomics and Molecular Aspects.

[109] Grönlund M.M., Gueimonde M., Laitinen K., Kociubinski G., Grönroos T., Salminen S., Isolauri E. (2007). Maternal breast-milk and intestinal bifidobacteria guide the compositional development of the Bifidobacterium microbiota in infants at risk of allergic disease. Clin. Exp. Allergy.

[110] Menard O., Butel M.-J., Gaboriau-Routhiau V., Waligora-Dupriet A.-J. (2008). Gnotobiotic mouse immune response induced by Bifidobacterium sp. strains isolated from infants. Appl. Environ. Microbiol..

[111] Young S.L., Simon M.A., Baird M.A., Tannock G.W., Bibiloni R., Spencely K., Lane J.M., Fitzharris P., Crane J., Town I. (2004). Bifidobacterial species differentially affect expression of cell surface markers and cytokines of dendritic cells harvested from cord blood. Clin. Vaccine Immunol..

[112] Collado M.C., Isolauri E., Laitinen K., Salminen S. (2008). Distinct composition of gut microbiota during pregnancy in overweight and normal-weight women. Am. J. Clin. Nutr..

[113] Ley R.E., Bäckhed F., Turnbaugh P., Lozupone C.A., Knight R.D., Gordon J.I. (2005). Obesity alters gut microbial ecology. Proc. Natl. Acad. Sci. USA.

[114] Zhang H., DiBaise J.K., Zuccolo A., Kudrna D., Braidotti M., Yu Y., Parameswaran P., Crowell M.D., Wing R., Rittmann B.E. (2009). Human gut microbiota in obesity and after gastric bypass. Proc. Natl. Acad. Sci. USA.

[115] Bäckhed F., Manchester J.K., Semenkovich C.F., Gordon J.I. (2007). Mechanisms underlying the resistance to diet-induced obesity in germ-free mice. Proc. Natl. Acad. Sci. USA.

[116] Larsen N., Vogensen F.K., Van Den Berg F.W., Nielsen D.S., Andreasen A.S., Pedersen B.K., Al-Soud W.A., Sørensen S.J., Hansen L.H., Jakobsen M. (2010). Gut microbiota in human adults with type 2 diabetes differs from non-diabetic adults. PLoS ONE.

[117] Seksik P., Rigottier-Gois L., Gramet G., Sutren M., Pochart P., Marteau P., Jian R., Doré J. (2003). Alterations of the dominant faecal bacterial groups in patients with Crohn’s disease of the colon. Gut.

[118] Wang T., Cai G., Qiu Y., Fei N., Zhang M., Pang X., Jia W., Cai S., Zhao L. (2012). Structural segregation of gut microbiota between colorectal cancer patients and healthy volunteers. ISME J..

[119] Krogius-Kurikka L., Lyra A., Malinen E., Aarnikunnas J., Tuimala J., Paulin L., Mäkivuokko H., Kajander K., Palva A. (2009). Microbial community analysis reveals high level phylogenetic alterations in the overall gastrointestinal microbiota of diarrhoea-predominant irritable bowel syndrome sufferers. BMC Gastroenterol..

[120] Fei N., Zhao L. (2013). An opportunistic pathogen isolated from the gut of an obese human causes obesity in germfree mice. ISME J..

[121] Britton G.J., Contijoch E.J., Mogno I., Vennaro O.H., Llewellyn S.R., Ng R., Li Z., Mortha A., Merad M., Das A. (2019). Microbiotas from humans with inflammatory bowel disease alter the balance of gut Th17 and RORγt+ regulatory T cells and exacerbate colitis in mice. Immunity.

[122] Zheng P., Zeng B., Zhou C., Liu M., Fang Z., Xu X., Zeng L., Chen J., Fan S., Du X. (2016). Gut microbiome remodeling induces depressive-like behaviors through a pathway mediated by the host’s metabolism. Mol. Psychiatry.

[123] Henke M.T., Kenny D.J., Cassilly C.D., Vlamakis H., Xavier R.J., Clardy J. (2019). Ruminococcus gnavus, a member of the human gut microbiome associated with Crohn’s disease, produces an inflammatory polysaccharide. Proc. Natl. Acad. Sci. USA.

[124] De Filippis F., Paparo L., Nocerino R., Della Gatta G., Carucci L., Russo R., Pasolli E., Ercolini D., Berni Canani R. (2021). Specific gut microbiome signatures and the associated pro-inflamatory functions are linked to pediatric allergy and acquisition of immune tolerance. Nat. Commun..

[125] Ruiz-Saavedra S., Arboleya S., Nogacka A.M., González del Rey C., Suárez A., Diaz Y., Gueimonde M., Salazar N., González S., de Los Reyes-Gavilán C.G. (2023). Commensal Fecal Microbiota Profiles Associated with Initial Stages of Intestinal Mucosa Damage: A Pilot Study. Cancers.

[126] Valentino V., De Filippis F., Marotta R., Pasolli E., Ercolini D. (2024). Genomic features and prevalence of Ruminococcus species in humans are associated with age, lifestyle, and disease. Cell Rep..

[127] Dahl C., Stanislawski M., Iszatt N., Mandal S., Lozupone C., Clemente J.C., Knight R., Stigum H., Eggesbø M. (2017). Gut microbiome of mothers delivering prematurely shows reduced diversity and lower relative abundance of Bifidobacterium and Streptococcus. PLoS ONE.

[128] Davies J., Mayer M.J., Juge N., Narbad A., Sayavedra L. (2024). Bacteroides thetaiotaomicron enhances H2S production in Bilophila wadsworthia. Gut Microbes.

[129] Feng Z., Long W., Hao B., Ding D., Ma X., Zhao L., Pang X. (2017). A human stool-derived Bilophila wadsworthia strain caused systemic inflammation in specific-pathogen-free mice. Gut Pathog..

[130] Natividad J.M., Lamas B., Pham H.P., Michel M.-L., Rainteau D., Bridonneau C., Da Costa G., van Hylckama Vlieg J., Sovran B., Chamignon C. (2018). Bilophila wadsworthia aggravates high fat diet induced metabolic dysfunctions in mice. Nat. Commun..

[131] Dordević D., Jančíková S., Vítězová M., Kushkevych I. (2021). Hydrogen sulfide toxicity in the gut environment: Meta-analysis of sulfate-reducing and lactic acid bacteria in inflammatory processes. J. Adv. Res..

[132] Li G., Yin P., Chu S., Gao W., Cui S., Guo S., Xu Y., Yuan E., Zhu T., You J. (2021). Correlation analysis between GDM and gut microbial composition in late pregnancy. J. Diabetes Res..

[133] Fu F., Wang F., Ding J., Xiao L., Song X. (2023). Effects of Weight Gain During Pregnancy in Normal-Weight Women on Changes in the Gut Microbiota of Pregnant Women in the Third Trimester. Biochem. Genet..

[134] Ma G., Yan H., Tye K.D., Tang X., Luo H., Li Z., Xiao X. (2024). Effect of probiotic administration during pregnancy on the functional diversity of the gut microbiota in healthy pregnant women. Microbiol. Spectr..

[135] Kuang Y.-S., Lu J.-H., Li S.-H., Li J.-H., Yuan M.-Y., He J.-R., Chen N.-N., Xiao W.-Q., Shen S.-Y., Qiu L. (2017). Connections between the human gut microbiome and gestational diabetes mellitus. Gigascience.

[136] Cortez R.V., Taddei C.R., Sparvoli L.G., Ângelo A.G., Padilha M., Mattar R., Daher S. (2019). Microbiome and its relation to gestational diabetes. Endocrine.

[137] Crusell M.K.W., Hansen T.H., Nielsen T., Allin K.H., Rühlemann M.C., Damm P., Vestergaard H., Rørbye C., Jørgensen N.R., Christiansen O.B. (2018). Gestational diabetes is associated with change in the gut microbiota composition in third trimester of pregnancy and postpartum. Microbiome.

[138] Liu H., Pan L.-L., Lv S., Yang Q., Zhang H., Chen W., Lv Z., Sun J. (2019). Alterations of gut microbiota and blood lipidome in gestational diabetes mellitus with hyperlipidemia. Front. Physiol..

[139] Wu Y., Bible P.W., Long S., Ming W.-k., Ding W., Long Y., Wen X., Li X., Deng X., Deng Y. (2020). Metagenomic analysis reveals gestational diabetes mellitus-related microbial regulators of glucose tolerance. Acta Diabetol..

[140] Ye G., Zhang L., Wang M., Chen Y., Gu S., Wang K., Leng J., Gu Y., Xie X. (2019). The gut microbiota in women suffering from gestational diabetes mellitus with the failure of glycemic control by lifestyle modification. J. Diabetes Res..

[141] Xu Y., Zhang M., Zhang J., Sun Z., Ran L., Ban Y., Wang B., Hou X., Zhai S., Ren L. (2020). Differential intestinal and oral microbiota features associated with gestational diabetes and maternal inflammation. Am. J. Physiol.-Endocrinol. Metab..

[142] Qin J., Li Y., Cai Z., Li S., Zhu J., Zhang F., Liang S., Zhang W., Guan Y., Shen D. (2012). A metagenome-wide association study of gut microbiota in type 2 diabetes. Nature.

[143] Karlsson F.H., Tremaroli V., Nookaew I., Bergström G., Behre C.J., Fagerberg B., Nielsen J., Bäckhed F. (2013). Gut metagenome in European women with normal, impaired and diabetic glucose control. Nature.

[144] Chen P.-C., Chien Y.-W., Yang S.-C. (2019). The alteration of gut microbiota in newly diagnosed type 2 diabetic patients. Nutrition.

[145] Shih C.-T., Yeh Y.-T., Lin C.-C., Yang L.-Y., Chiang C.-P. (2020). Akkermansia muciniphila is negatively correlated with hemoglobin A1c in refractory diabetes. Microorganisms.

[146] Pinto Y., Frishman S., Turjeman S., Eshel A., Nuriel-Ohayon M., Shtossel O., Ziv O., Walters W., Parsonnet J., Ley C. (2023). Gestational diabetes is driven by microbiota-induced inflammation months before diagnosis. Gut.

[147] Mullins T.P., Tomsett K.I., Gallo L.A., Callaway L.K., McIntyre H.D., Nitert M.D., Barrett H.L. (2021). Maternal gut microbiota displays minor changes in overweight and obese women with GDM. Nutr. Metab. Cardiovasc. Dis..

[148] Chen Z., Radjabzadeh D., Chen L., Kurilshikov A., Kavousi M., Ahmadizar F., Ikram M.A., Uitterlinden A.G., Zhernakova A., Fu J. (2021). Association of insulin resistance and type 2 diabetes with gut microbial diversity: A microbiome-wide analysis from population studies. JAMA Netw. Open.

[149] Le Chatelier E., Nielsen T., Qin J., Prifti E., Hildebrand F., Falony G., Almeida M., Arumugam M., Batto J.-M., Kennedy S. (2013). Richness of human gut microbiome correlates with metabolic markers. Nature.

[150] Huang L., Sililas P., Thonusin C., Luewan S., Chattipakorn S.C. (2021). Early gut dysbiosis could be an indicator of unsuccessful diet control in gestational diabetes mellitus. J. Diabetes.

[151] Wu N., Zhou J., Mo H., Mu Q., Su H., Li M., Yu Y., Liu A., Zhang Q., Xu J. (2022). The gut microbial signature of gestational diabetes mellitus and the association with diet intervention. Front. Cell. Infect. Microbiol..

[152] Turnbaugh P.J., Ley R.E., Mahowald M.A., Magrini V., Mardis E.R., Gordon J.I. (2006). An obesity-associated gut microbiome with increased capacity for energy harvest. Nature.

[153] Bahar-Tokman H., Demirci M., Keskin F.E., Cagatay P., Taner Z., Ozturk-Bakar Y., Ozyazar M., Kiraz N., Kocazeybek B.S. (2022). Firmicutes/Bacteroidetes Ratio in the Gut Microbiota and IL-1β, IL-6, IL-8, TLR2, TLR4, TLR5 Gene Expressions in Type 2 Diabetes. Clin. Lab..

[154] Mousavi S.N., Momeni N., Chiti H., Mahmoodnasab H., Ahmadi M., Heidarzadeh S. (2025). Higher gut Bacteroidetes and Actinobacteria population in early pregnancy is associated with lower risk of gestational diabetes in the second trimester. BMC Pregnancy Childbirth.

[155] Salamon D., Sroka-Oleksiak A., Kapusta P., Szopa M., Mrozińska S., Ludwig-Słomczyńska A.H., Wołkow P.P., Bulanda M., Klupa T., Małecki M.T. (2018). Characteristics of gut microbiota in adult patients with type 1 and type 2 diabetes based on next-generation sequencing of the 16S rRNA gene fragment. Pol. Arch. Intern. Med..

[156] Zhang X., Shen D., Fang Z., Jie Z., Qiu X., Zhang C., Chen Y., Ji L. (2013). Human gut microbiota changes reveal the progression of glucose intolerance. PLoS ONE.

[157] Huang S., Chen J., Cui Z., Ma K., Wu D., Luo J., Li F., Xiong W., Rao S., Xiang Q. (2023). Lachnospiraceae-derived butyrate mediates protection of high fermentable fiber against placental inflammation in gestational diabetes mellitus. Sci. Adv..

[158] Cui Z., Wang S., Niu J., Ma J., Yang H. (2024). Bifidobacterium species serve as key gut microbiome regulators after intervention in gestational diabetes mellitus. BMC Microbiol..

[159] O’Callaghan A., Van Sinderen D. (2016). Bifidobacteria and their role as members of the human gut microbiota. Front. Microbiol..

[160] Wang C., Zhang H., Liu H., Zhang H., Bao Y., Di J., Hu C. (2020). The genus Sutterella is a potential contributor to glucose metabolism improvement after Roux-en-Y gastric bypass surgery in T2D. Diabetes Res. Clin. Pract..

[161] Su M., Nie Y., Shao R., Duan S., Jiang Y., Wang M., Xing Z., Sun Q., Liu X., Xu W. (2018). Diversified gut microbiota in newborns of mothers with gestational diabetes mellitus. PLoS ONE.

[162] Nagpal R., Wang S., Solberg Woods L.C., Seshie O., Chung S.T., Shively C.A., Register T.C., Craft S., McClain D.A., Yadav H. (2018). Comparative microbiome signatures and short-chain fatty acids in mouse, rat, non-human primate, and human feces. Front. Microbiol..

[163] Huang X., Yu Y., Tian N., Huang J., Zhang X., Yu R. (2025). Human microbiota-associated animal models: A review. Front. Cell. Infect. Microbiol..

[164] Bertorello S., Cei F., Fink D., Niccolai E., Amedei A. (2024). The future exploring of gut microbiome-immunity interactions: From in vivo/vitro models to in silico innovations. Microorganisms.

[165] Weingarden A.R. (2026). Strengths and limitations of in vitro and animal models to advance understanding of human diet-microbiome interactions. Gut Microbes Rep..

[166] Chu M., Zhang X. (2022). Bacterial atlas of mouse gut microbiota. Cell. Microbiol..

[167] Livanos A.E., Greiner T.U., Vangay P., Pathmasiri W., Stewart D., McRitchie S., Li H., Chung J., Sohn J., Kim S. (2016). Antibiotic-mediated gut microbiome perturbation accelerates development of type 1 diabetes in mice. Nat. Microbiol..

[168] Zhu Y., He C., Li X., Cai Y., Hu J., Liao Y., Zhao J., Xia L., He W., Liu L. (2019). Gut microbiota dysbiosis worsens the severity of acute pancreatitis in patients and mice. J. Gastroenterol..

[169] Zhan G., Yang N., Li S., Huang N., Fang X., Zhang J., Zhu B., Yang L., Yang C., Luo A. (2018). Abnormal gut microbiota composition contributes to cognitive dysfunction in SAMP8 mice. Aging.

[170] Yu F., Han W., Zhan G., Li S., Xiang S., Zhu B., Jiang X., Yang L., Luo A., Hua F. (2019). Abnormal gut microbiota composition contributes to cognitive dysfunction in streptozotocin-induced diabetic mice. Aging.

[171] Gohir W., Whelan F.J., Surette M.G., Moore C., Schertzer J.D., Sloboda D.M. (2015). Pregnancy-related changes in the maternal gut microbiota are dependent upon the mother’s periconceptional diet. Gut Microbes.

[172] Jašarević E., Howard C.D., Misic A.M., Beiting D.P., Bale T.L. (2017). Stress during pregnancy alters temporal and spatial dynamics of the maternal and offspring microbiome in a sex-specific manner. Sci. Rep..

[173] Faas M.M., Liu Y., Borghuis T., van Loo-Bouwman C.A., Harmsen H., De Vos P. (2020). Microbiota induced changes in the immune response in pregnant mice. Front. Immunol..

[174] Khan I., Azhar E.I., Abbas A.T., Kumosani T., Barbour E.K., Raoult D., Yasir M. (2016). Metagenomic analysis of antibiotic-induced changes in gut microbiota in a pregnant rat model. Front. Pharmacol..

[175] Cordero-Varela J.A., Reyes-Corral M., Lao-Pérez M., Fernández-Santos B., Montenegro-Elvira F., Sempere L., Ybot-González P. (2023). Analysis of Gut Characteristics and Microbiota Changes with Maternal Supplementation in a Neural Tube Defect Mouse Model. Nutrients.

[176] Yao Z., Long Y., Ye J., Li P., Jiang Y., Chen Y. (2020). 16S rRNA gene-based analysis reveals the effects of gestational diabetes on the gut microbiota of mice during pregnancy. Indian J. Microbiol..

[177] Mu Q., Cabana-Puig X., Mao J., Swartwout B., Abdelhamid L., Cecere T.E., Wang H., Reilly C.M., Luo X.M. (2019). Pregnancy and lactation interfere with the response of autoimmunity to modulation of gut microbiota. Microbiome.

[178] Aizawa S., Uebanso T., Shimohata T., Mawatari K., Takahashi A. (2023). Effects of the loss of maternal gut microbiota before pregnancy on gut microbiota, food allergy susceptibility, and epigenetic modification on subsequent generations. Biosci. Microbiota Food Health.

[179] Caesar R., Reigstad C.S., Bäckhed H.K., Reinhardt C., Ketonen M., Lundén G.Ö., Cani P.D., Bäckhed F. (2012). Gut-derived lipopolysaccharide augments adipose macrophage accumulation but is not essential for impaired glucose or insulin tolerance in mice. Gut.

[180] Elderman M., Hugenholtz F., Belzer C., Boekschoten M., de Haan B., de Vos P., Faas M. (2018). Changes in intestinal gene expression and microbiota composition during late pregnancy are mouse strain dependent. Sci. Rep..

[181] Younge N., McCann J.R., Ballard J., Plunkett C., Akhtar S., Araújo-Pérez F., Murtha A., Brandon D., Seed P.C. (2019). Fetal exposure to the maternal microbiota in humans and mice. JCI Insight.

[182] Kondo S., Xiao J.-z., Satoh T., Odamaki T., Takahashi S., Sugahara H., Yaeshima T., Iwatsuki K., Kamei A., Abe K. (2010). Antiobesity effects of Bifidobacterium breve strain B-3 supplementation in a mouse model with high-fat diet-induced obesity. Biosci. Biotechnol. Biochem..

[183] Kikuchi K., Othman M.B., Sakamoto K. (2018). Sterilized bifidobacteria suppressed fat accumulation and blood glucose level. Biochem. Biophys. Res. Commun..

[184] Le T.K.C., Hosaka T., Le T.T.T., Nguyen T.G., Tran Q.B., Le T.H.H., Da Pham X. (2014). Oral administration of Bifidobacterium spp. improves insulin resistance, induces adiponectin, and prevents inflammatory adipokine expressions. Biomed. Res..

[185] Kim S.H., Huh C.S., Choi I.D., Jeong J.W., Ku H.K., Ra J.H., Kim T.Y., Kim G.B., Sim J.H., Ahn Y.T. (2014). The anti-diabetic activity of Bifidobacterium lactis HY8101 in vitro and in vivo. J. Appl. Microbiol..

[186] Caruso R., Mathes T., Martens E., Kamada N., Nusrat A., Inohara N., Núñez G. (2019). A specific gene-microbe interaction drives the development of Crohn’s disease–like colitis in mice. Sci. Immunol..

[187] Villa C.R., Taibi A., Kasee S., Chen J., Ward W.E., Comelli E.M. (2017). Low Dietary Vitamin D since Pre-mating Does not Modify Fecal Bacteroides Counts of Mouse Dams at the End of Pregnancy. FASEB J..

[188] Gurung M., Li Z., You H., Rodrigues R., Jump D.B., Morgun A., Shulzhenko N. (2020). Role of gut microbiota in type 2 diabetes pathophysiology. EBioMedicine.

